# Supplementary material for: Transcriptome Analysis Reveals Cross-Talk between the Flagellar Transcriptional Hierarchy and Secretion System in Plesiomonas shigelloides
Source: Int J Mol Sci. 2024 Jul 5;25(13):7375. doi: 10.3390/ijms25137375 (PMC11242183; doi:10.3390/ijms25137375)
Supplement: Supplementary file 1 [file ijms-25-07375-s001.zip › ijms-3071077-SI.pdf]

Table S1 Bacterial strains and plasmids used in this study.

Strains/plasmids	Genotype or relevant characteristics[a]	Source or reference
Plesiomonas shigelloides strains		
G5884	Wild type, serotype O45:H2[b]; Amp[r]	CNCTC[b] Aer 44/89
Δ*flaK*	*flaK* gene deletion mutant of G5884; Amp[r]	This study
Δ*flaM*	*flaM* gene deletion mutant of G5884; Amp[r]	This study
Δ*fliA*	*fliA* gene deletion mutant of G5884; Amp[r]	This study
Δ*fliA*$_L$	*fliA*$_L$ gene deletion mutant of G5884; Amp[r]	This study
pBAD33/*flaK*$^+$	Δ*flaK* containing pBAD33 carrying *flaK* ORF with its own promoter	This study
pBAD33/*flaM*$^+$	Δ*flaM* containing pBAD33 carrying *flaM* ORF with its own promoter	This study
pBAD33/*fliA*$^+$	Δ*fliA* containing pBAD33 carrying *flaM* ORF with its own promoter	This study
pBAD33/*fliA*$_L^+$	Δ*fliA*$_L$ containing pBAD33 carrying *fliA*$_L$ ORF with its own promoter	This study
WT/lux	WT containing pMS402; Amp[r], Km[r]	This study
WT/*flhA*-lux	WT containing pMS402 carrying *flhA* promoter; Amp[r], Km[r]	This study
WT/*flhB*-lux	WT containing pMS402 carrying *flhB* promoter; Amp[r], Km[r]	This study

WT/*fliE*-lux	WT containing pMS402 carrying *fliE* promoter; Amp^r, Km^r	This study
WT/*fliK*-lux	WT containing pMS402 carrying *fliK* promoter; Amp^r, Km^r	This study
WT/*cheY*-lux	WT containing pMS402 carrying *cheY* promoter; Amp^r, Km^r	This study
WT/*flaM*-lux	WT containing pMS402 carrying *flaM* promoter; Amp^r, Km^r	This study
WT/*fliA$_L$*-lux	WT containing pMS402 carrying *fliA$_L$* promoter; Amp^r, Km^r	This study
WT/*flgA*-lux	WT containing pMS402 carrying *flgA* promoter; Amp^r, Km^r	This study
WT/*flgM*-lux	WT containing pMS402 carrying *flgM* promoter; Amp^r, Km^r	This study
WT/*flgB*-lux	WT containing pMS402 carrying *flgB* promoter; Amp^r, Km^r	This study
WT/*flgK*-lux	WT containing pMS402 carrying *flgK* promoter; Amp^r, Km^r	This study
WT/*flgO*-lux	WT containing pMS402 carrying *flgO* promoter; Amp^r, Km^r	This study
WT/*flaG*-lux	WT containing pMS402 carrying *flaG* promoter; Amp^r, Km^r	This study

WT/*cheV*-lux	WT containing pMS402 carrying *cheV* promoter; Amp^r, Km^r	This study
WT/*motA*-lux	WT containing pMS402 carrying *motA* promoter; Amp^r, Km^r	This study
WT/*pomA*-lux	WT containing pMS402 carrying *pomA* promoter; Amp^r, Km^r	This study
WT/*flaC*-lux	WT containing pMS402 carrying *flaC* promoter; Amp^r, Km^r	This study
WT/$fliM_L$-lux	WT containing pMS402 carrying $fliM_L$ promoter; Amp^r, Km^r	This study
WT/$flgB_L$-lux	WT containing pMS402 carrying $flgB_L$ promoter; Amp^r, Km^r	This study
WT/$fliE_L$-lux	WT containing pMS402 carrying $fliE_L$ promoter; Amp^r, Km^r	This study
WT/$flgD_L$-lux	WT containing pMS402 carrying $flgD_L$ promoter; Amp^r, Km^r	This study
WT/$flgA_L$-lux	WT containing pMS402 carrying $flgA_L$ promoter; Amp^r, Km^r	This study
WT/$flgK_L$-lux	WT containing pMS402 carrying $flgK_L$ promoter; Amp^r, Km^r	This study
WT/$flgM_L$-lux	WT containing pMS402 carrying $flgM_L$ promoter; Amp^r, Km^r	This study

WT/*fliC*-lux	WT containing pMS402 carrying *fliC* promoter; Amp^r, Km^r	This study
Δ*flaK/flhA*-lux	Δ*flaK* containing pMS402 carrying *flhA* promoter; Amp^r, Km^r	This study
Δ*flaK/flhB*-lux	Δ*flaK* containing pMS402 carrying *flhB* promoter; Amp^r, Km^r	This study
Δ*flaK/fliE*-lux	Δ*flaK* containing pMS402 carrying *fliE* promoter; Amp^r, Km^r	This study
Δ*flaK/fliK*-lux	Δ*flaK* containing pMS402 carrying *fliK* promoter; Amp^r, Km^r	This study
Δ*flaK/cheY*-lux	Δ*flaK* containing pMS402 carrying *cheY* promoter; Amp^r, Km^r	This study
Δ*flaK/flaM*-lux	Δ*flaK* containing pMS402 carrying *flaM* promoter; Amp^r, Km^r	This study
Δ*flaK*/$fliA_L$-lux	Δ*flaK* containing pMS402 carrying $fliA_L$ promoter; Amp^r, Km^r	This study
Δ*flaM/flgA*-lux	Δ*flaM* containing pMS402 carrying *flgA* promoter; Amp^r, Km^r	This study
Δ*flaM/flgM*-lux	Δ*flaM* containing pMS402 carrying *flgM* promoter; Amp^r, Km^r	This study
Δ*flaM/flgB*-lux	Δ*flaM* containing pMS402 carrying *flgB* promoter; Amp^r, Km^r	This study

Δ*flaM/flgK*-lux	Δ*flaM* containing pMS402 carrying *flgK* promoter; Amp^r, Km^r	This study
Δ*flaM/flgO*-lux	Δ*flaM* containing pMS402 carrying *flgO* promoter; Amp^r, Km^r	This study
Δ*fliA/flaG*-lux	Δ*fliA* containing pMS402 carrying *flaG* promoter; Amp^r, Km^r	This study
Δ*fliA/cheV*-lux	Δ*fliA* containing pMS402 carrying *cheV* promoter; Amp^r, Km^r	This study
Δ*fliA/motA*-lux	Δ*fliA* containing pMS402 carrying *motA* promoter; Amp^r, Km^r	This study
Δ*fliA/pomA*-lux	Δ*fliA* containing pMS402 carrying *pomA* promoter; Amp^r, Km^r	This study
Δ*fliA/flaC*-lux	Δ*fliA* containing pMS402 carrying *flaC* promoter; Amp^r, Km^r	This study
Δ*flaK/fliM$_L$*-lux	Δ*flaK* containing pMS402 carrying *fliM$_L$* promoter; Amp^r, Km^r	This study
Δ*flaK/flgB$_L$*-lux	Δ*flaK* containing pMS402 carrying *flgB$_L$* promoter; Amp^r, Km^r	This study
Δ*flaK/fliE$_L$*-lux	Δ*flaK* containing pMS402 carrying *fliE$_L$* promoter; Amp^r, Km^r	This study
Δ*flaK/flgD$_L$*-lux	Δ*flaK* containing pMS402 carrying *flgD$_L$* promoter; Amp^r, Km^r	This study

Δ*fliA*$_L$/*flgA*$_L$-lux	Δ*fliA*$_L$ containing pMS402 carrying *flgA*$_L$ promoter; Amp^r, Km^r	This study
Δ*fliA*$_L$/*flgK*$_L$-lux	Δ*fliA*$_L$ containing pMS402 carrying *flgK*$_L$ promoter; Amp^r, Km^r	This study
Δ*fliA*$_L$/*flgM*$_L$-lux	Δ*fliA*$_L$ containing pMS402 carrying *flgM*$_L$ promoter; Amp^r, Km^r	This study
Δ*fliA*$_L$/*fliC*-lux	Δ*fliA*$_L$ containing pMS402 carrying *fliC* promoter; Amp^r, Km^r	This study
E. coli strains		
DH5α λ*pir*	Transformation host	Lab collection
S17–1 λ*pir*	Tp^R Sm^R *recA*, *thi*, *pro*, *hsdR*-M$^+$RP4: 2-Tc:Mu: Km Tn7 λ*pir*, Km^r , Sm^r , Tp^r	Lab collection
BL21(DE3)	Host strain for protein expression	Lab collection
MG1655	F-λ-ilvG-rfb-50 rph-1; Cm^r	Lab collection
BL21/pMAL-c5X-*flaK*	BL21 with pMAL-c5X carrying the *flaK* gene; Amp^r	This study
BL21/pMAL-c5X-*flaM*	BL21 with pMAL-c5X carrying the *flaM* gene; Amp^r	This study
BL21/pMAL-c5X-*fliA*	BL21 with pMAL-c5X carrying the *fliA* gene; Amp^r	This study
BL21/pMAL-c5X-*fliA*$_L$	BL21 with pMAL-c5X carrying the *fliA*$_L$ gene; Amp^r	This study
Plasmids		
pRE112	Widely used gene knocked vector, with onT RP4; Cm^r	Lab collection

pMS402	For construct promoter-luxCDABE reporter fusion; Km^r	Lab collection
pBAD33	Arabinose inducible expression vector; Cm^r	Lab collection
pRE112-*flaK*$^-$	pRE112 containing the homologous arms of *flaK* gene of G5884; Cm^r	This study
pRE112-*flaM*$^-$	pRE112 containing the homologous arms of *flaM* gene of G5884; Cm^r	This study
pRE112-*fliA*$^-$	pRE112 containing the homologous arms of *fliA* gene of G5884; Cm^r	This study
pRE112-$fliA_L^-$	pRE112 containing the homologous arms of $fliA_L$ gene of G5884; Cm^r	This study
pBAD33-*flaK*$^+$	pBAD33 with complete *flaK*; Cm^r	This study
pBAD33-*flaM*$^+$	pBAD33 with complete *flaM*; Cm^r	This study
pBAD33-*fliA*$^+$	pBAD33 with complete *fliA*; Cm^r	This study
pBAD33-$fliA_L^+$	pBAD33 with complete $fliA_L$; Cm^r	This study
pMS402-*flhA*	pMS402 with *flhA* promoter; Kmr	This study
pMS402-*flhB*	pMS402 with *flhB* promoter; Kmr	This study
pMS402-*fliE*	pMS402 with *fliE* promoter; Kmr	This study
pMS402-*fliK*	pMS402 with *fliK* promoter; Kmr	This study
pMS402-*cheY*	pMS402 with *cheY* promoter; Kmr	This study

pMS402-*flaM*	pMS402 with *flaM* promoter; Kmr	This study
pMS402-*fliA_L*	pMS402 with *fliA_L* promoter; Kmr	This study
pMS402-*flgA*	pMS402 with *flgA* promoter; Kmr	This study
pMS402-*flgM*	pMS402 with *flgM* promoter; Kmr	This study
pMS402-*flgB*	pMS402 with *flgB* promoter; Kmr	This study
pMS402-*flgK*	pMS402 with *flgK* promoter; Km^r	This study
pMS402-*flgO*	pMS402 with *flgO* promoter; Km^r	This study
pMS402-*flgT*	pMS402 with *flgT* promoter; Km^r	This study
pMS402-*flaG*	pMS402 with *flaG* promoter; Km^r	This study
pMS402-*cheV*	pMS402 with *cheV* promoter; Km^r	This study
pMS402-*motA*	pMS402 with *motA* promoter; Km^r	This study
pMS402-*pomA*	pMS402 with *pomA* promoter; Km^r	This study
pMS402-*flaC*	pMS402 with *flaC* promoter; Km^r	This study
pMS402-$fliM_L$	pMS402 with $fliM_L$ promoter; Km^r	This study
pMS402-$flgB_L$	pMS402 with $flgB_L$ promoter; Km^r	This study

pMS402-*fliE*$_L$	pMS402 with *fliE*$_L$ promoter; Km^r	This study
pMS402-*fliD*$_L$	pMS402 with *fliD*$_L$ promoter; Km^r	This study
pMS402-*flgA*$_L$	pMS402 with *flgA*$_L$ promoter; Km^r	This study
pMS402-*flgK*$_L$	pMS402 with *flgK*$_L$ promoter; Km^r	This study
pMS402-*flgM*$_L$	pMS402 with *flgM*$_L$ promoter; Km^r	This study
pMS402-*fliC*$_L$	pMS402 with *fliC*$_L$ promoter; Km^r	This study

[a] r = resistant.

[b] CNCTC, Czech National Collection of Type Cultures, the Czech Republic.

Table S2 Primers used in this study

Name	Sequence (5′–3′)
Primers for construction of mutants	
Δ*flaK*-F1	GCTCTAGAGGTGAGCGTCTCGGGAAG
Δ*flaK*-R1	GGAGCAGGAGCGATTCTAGTATTTATGGCAGCG
Δ*flaK*-F2	ACTAGAATCGCTCCTGCTCCTAAACCCAAA
Δ*flaK*-R2	GGGGTACCCTCATATCGCATGCCAACG
Δ*flaK*-P1	GGTGAGCGTCTCGGGAAG
Δ*flaK*-P4	CTCATATCGCATGCCAACG
Δ*flaM*-F1	GCTCTAGAAAGACGAGAAGGCCTACGC
Δ*flaM*-R1	CGATGCGGGACTGAAAACAGTGATTTATGGAGAAC

Δ*flaM*-F2	CTGTTTTCAGTCCCGCATCGCATTTTT
Δ*flaM*-R2	GGGGTACCGCCGTCAGACAGGCATACTC
Δ*flaM*-P1	AAGACGAGAAGGCCTACGC
Δ*flaM*-P4	GCCGTCAGACAGGCATACTC
Δ*fliA*-F1	GCTCTAGAAAGATGTGCTGGTTACCGTGT
Δ*fliA*-R1	AAACGTGCCGAGGGCTTTACTCACGACACTCT
Δ*fliA*-F2	GTAAAGCCCTCGGCACGTTTTATTCACTCA
Δ*fliA*-R2	GGGGTACCCAGCTCTTTGGCTTGTTCG
Δ*fliA*-P1	AAGATGTGCTGGTTACCGTGT
Δ*fliA*-P4	CAGCTCTTTGGCTTGTTCG
Δ$fliA_L$-F1	GCTCTAGAAGAGAGAACACGATGTCGCAA
Δ$fliA_L$-R1	ATACCCCCCAAGTCGGTTACTGCACCACCA
Δ$fliA_L$-F2	GTAACCGACTTGGGGGGTATATGCAACG
Δ$fliA_L$-R2	GGGGTACCGAGGGGCGCAACAGGT
Δ$fliA_L$-P1	AGAGAGAACACGATGTCGCAA
Δ$fliA_L$-P4	GAGGGGCGCAACAGGT
Primers for identification of plasmid	
pRE112-F	CACTGTTCGTCCATTTCCG
pRE112-R	TTCGTCTCAGCCAATCCCT
pBAD33-F	AACAAAGCGGGACCAAAG
pBAD33-R	AGAGCGTTCACCGACAAA
pMS402-F	GGTCAAATGAATGCAGGGCT
pMS402-R	AGAGTCATTCAATATTGGCAGGT
pMAL-c5X-F	GGTGAAATCATGCCGAACATCC

pMAL-c5X-R	TCGCAACGTTCAAATCCGCT
Primers for construction of complemented strain	
pBAD33-*flaK*$^+$-F	CGAGCTCATGCCGGGACTGCAAAAA
pBAD33-*flaK*$^+$-R	GCTCTAGATCAAACGGCTGCATTTTCC
pBAD33-*flaM*$^+$-F	GGGGTACCGTGCGACTTAAGCAAAATGAAG
pBAD33-*flaM*$^+$-R	GCTCTAGATTACTGCGGGATCGCTATC
pBAD33-*fliA*$^+$-F	GGGGTACCGTGAGTAAAGCCCTGGCTTACA
pBAD33-*fliA*$^+$-R	GCTCTAGATTATACGGCTGACCAAGCATG
pBAD33-$fliA_L^+$-F	GGGGTACCATGTGCGGAGCGGCG
pBAD33-$fliA_L^+$-R	GCTCTAGATTAGTCCGGCTGGTTCCAG
Primers for lux	
lux-*flhA*-F	CCGCTCGAGGCAAGCTGGCATCTCTGTAC
lux-*flhA*-R	CGGGATCCCTATCCTCTGTCTACCGCGC
lux-*flhB*-F	CCGCTCGAGTTTTGTTTCTCGGCTCGGTG
lux-*flhB*-R	CGGGATCCGCGCCTCCTTTACGGTTAC
lux-*fliE*-F	CCGCTCGAGGCAACGTCTTACCGGGTCTT
lux-*fliE*-R	CGGGATCCGCTGTGTGATACATGACGCTGA
lux-*fliK*-F	CCGCTCGAGTTGCTGCGCCGTTTACAAA
lux-*fliK*-R	CGGGATCCGAAGTTTGAGACACGGAGCC
lux-*cheY*-F	CCGCTCGAGAACGGCGGTAAAGTGATTGG
lux-*cheY*-R	CGGGATCCCGCTTCTTATACGGCTGACC
lux-*flaM*-F	CCGCTCGAGAGCGTGAAATACTCAATATGCCTAT
lux-*flaM*-R	CGGGATCCATGGAGAACTAAGCAAATAGCAGAC
lux-$fliA_L$-F	CCGCTCGAGGCAAGCTGGCATCTCTGTAC

lux-*fliA*$_L$-R	CGGGATCCCTATCCTCTGTCTACCGCGC
lux-*flgA*-F	CCGCTCGAGAGCACTTAGACGGATCATCGA
lux-*flgA*-R	CGGGATCCAACGTCTCCTGTTATGCAAAA
lux-*flgM*-F	CCGCTCGAGTCACTGTCTGTCGCTCTTGT
lux-*flgM*-R	CGGGATCCAATGTTGACGAGCTGGTTGG
lux-*flgB*-F	CCGCTCGAGTTTTGTTTCGGTCGCAGGTG
lux-*flgB*-R	CGGGATCCACTTAATGCCAAGACACCGC
lux-*flgK*-F	CCGCTCGAGGGTGGAAGTGATTCAGGTGC
lux-*flgK*-R	CGGGATCCTCCTCAATCTGATGTGCCCA
lux-*flgO*-F	CCGCTCGAGCGTAACGTTAGGGTAAAGCGG
lux-*flgO*-R	CGGGATCCGTAATGACATTGACACTCCGGC
lux-*flgT*-F	CCGCTCGAGACCAACTGACGCGCCATC
lux-*flgT*-R	CGGGATCCGCCGGAGTGTCAATGTCATT
lux-*flaG*-F	CCGCTCGAGGACATGTCAACTCAAGCCGG
lux-*flaG*-R	CGGGATCCGAATTAACCCAGCAGAGACAGC
lux-*cheV*-F	CCGCTCGAGGGTAATAGTTTGCCGTCCCG
lux-*cheV*-R	CGGGATCCAACGTGCTACTCCCAGGG
lux-*motA*-F	CCGCTCGAGTCCCCAGGTCTCAAAATCGT
lux-*motA*-R	CGGGATCCCATCAAACCTCTGTGCTCGT
lux-*pomA*-F	CCGCTCGAGGCCAATACAATACCACGTTCA
lux-*pomA*-R	CGGGATCCCGTATTTGCGTCCCCATAGC
lux-*flaC*-F	CCGCTCGAGATTTCTTGACATGCCGCGTT
lux-*flaC*-R	CGGGATCCATCTCCGTTAAACTTGCCGC
lux-*fliM*$_L$-F	CCGCTCGAGATGACGCTGACTCTCCGAAAT

lux-*fliM*$_L$-R	CGGGATCCGCTCACTCGGCAACGTCTTA
lux-*fliE*$_L$-F	CCGCTCGAGGCAACGTCTTACCGGGTCTT
lux-*fliE*$_L$-R	CGGGATCCGCTGTGTGATACATGACGCTGA
lux-*flgB*$_L$-F	CCGCTCGAGCCTCGCTCAGGCGCTG
lux-*flgB*$_L$-R	CGGGATCCGTCCCCCTGCAAACAACG
lux-*flgA*$_L$-F	CCGCTCGAGGACGAGCGATTAATCTGCCG
lux-*flgA*$_L$-R	CGGGATCCCGTCTCCTTTAATGCGCAGG
lux-*flgK*$_L$-F	CCGCTCGAGCGGAAGTGATTGACGGACAG
lux-*flgK*$_L$-R	CGGGATCCCTCAGTGTAAAAACACCCGACC
lux-*flgM*$_L$-F	CCGCTCGAGACCGATCCACAGGCGCT
lux-*flgM*$_L$-R	CGGGATCCCCGGCTACTGGCCTAACTTAA
lux-*fliC*$_L$-F	CCGCTCGAGGATTGAGCGTCAGCTGCAAG
lux-*fliC*$_L$-R	CGGGATCCCTTGTTACTGGTATTAGGCGGAA
lux-*fliD*$_L$-F	CCGCTCGAGGCAAGCTGGCATCTCTGTAC
lux-*fliD*$_L$-R	CGGGATCCCTATCCTCTGTCTACCGCGC
Primers for qRT-PCR	
gyrB-RT-F	GATTTGCGCACTGGGTAGCC
gyrB-RT-R	GCGGCTGTTTGGATCCATGG
iolC-RT-F	TCTTAATCACTTGCACCGCG
iolC-RT-R	CATTGAGCACATCCTGACCG
iolE-RT-F	GCTTCCTCAATGCGGTACTG
iolE-RT-R	CCTCGGCAAAGCGATGTAAA
iolB-RT-F	ACTCAAAATATCACGCCGGC
iolB-RT-R	CAGGCAAACGGGTTATCTCG

napF-RT-F	GACCAGTTTATCGCGGCGTC
napF-RT-R	GCAACGCGTACACTGATCGG
napA-RT-F	ATGTGGGTGGAGAAGGAAGG
napA-RT-R	CAGGCCAAACTTCATCCACC
napG-RT-F	CTTTGAAGCGGCCTGTATCC
napG-RT-R	CTCATGATCCAGCAACACCG
hybO-RT-F	GCTGTAAAGGCCCAGAAACC
hybO-RT-R	TTTTGTGAACGCGGAGTCTG
hybB-RT-F	GTTTGAAACTGCCGCCTGTA
hybB-RT-R	GAACCCATCGACGATTGGTG
iscR-RT-F	GAGCAAGGACCGGTATCACT
iscR-RT-R	CCTGACAGTCACCTTTCCCT
aceE-RT-F	TGGGTTCTGGCGCTATCCTG
aceE-RT-R	TCACAGCAGTCACGAACGGT
aceF-RT-F	AGCGTCTGGAATCCGGTCAG
aceF-RT-R	TGGATCCGACCCAGCTCAAC
aceA-RT-F	CCCATGTCTGACAGTGCTTG
aceA-RT-R	CCTCTAAGCCGGATCTGGAG
treC-RT-F	GATGGATGACGCGCAGATTT
treC-RT-R	GTGAAGATCGGATGCGCTTT
treB-RT-F	TATCACTGGCGGTCTGATCC
treB-RT-R	CCAACCGGCAAGAAATGGAA
hybC-RT-F	TATCGCCAGCTTCTACCCAG
hybC-RT-R	CTTGCTACTTTCGGCGATCC

hybA-RT-F	TTCACAAGTGTCAGCTGTGC
hybA-RT-R	GATCATGGCTGTTCACGGTC
napB-RT-F	CTTCCCTGATGGCGAATGTG
napB-RT-R	GTGGTGCGGTAGTTCTCAAC
napC-RT-F	CGGTTTTATTGCTGGCGTCT
napC-RT-R	TACGCACCATCTTAGGCACA
glmU-RT-F	TCCATCTGATCTACGGCCAC
glmU-RT-R	TTCGCCGTCAACAAACCAAT
flhB-RT-F	GTGGTTTGTTCTGCTGCTCA
flhB-RT-R	TGCTCAGGGTTGGTGATGAT
flhA-RT-F	TACGCTGTGGGTATTGTGGT
flhA-RT-R	TCCATGGAGCCGTAGAAGTC
flhF-RT-F	TGGTGCCCATGAACAATTGG
flhF-RT-R	CTGTTCTGTCAGGCGCAAAT
flhG-RT-F	GATGTGCTGGTTACCGTGTG
flhG-RT-R	CCAACCGGACATGTTCATCC
fliA-RT-F	GCATTACGGAAGCGATCGAA
fliA-RT-R	CCACGCCTTGAAACGGTAAA
fliE-RT-F	AGTGCTTTGGACTTCTCTGCT
fliE-RT-R	TACCTGTACCGTCGCCTCAA
fliF-RT-F	TGAGCAAATTCGCAACGTGA
fliF-RT-R	CTTGGCTACCGGAATTCAGC
fliG-RT-F	GCCATGACTTCGTGCGTAAT
fliG-RT-R	CACAATGGTCTGGATCTGCG

fliH-RT-F	GGCTATAACCGCACAATCCG
fliH-RT-R	AGCCTTCTTGTTTGCCTTCG
fliI-RT-F	CCGCGTTCTTTACCGTACTG
fliI-RT-R	TCCTGATTGCGGTTGTAGGT
fliJ-RT-F	CTGCAGCAGTTTATTGGCCA
fliJ-RT-R	CGCCGCCATCTCATCATTTA
fliK-RT-F	GCGAGTTACTGGAGCAAACC
fliK-RT-R	TACCAACATCGCTGCCATTG
fliL-RT-F	TACTACTGGTTTGCTGCGGA
fliL-RT-R	GATCGGTTTCACTGCGTACC
fliM-RT-F	GCCGACTAGCTTGAACATGG
fliM-RT-R	TCCCCACGCCTCTTTGTAAT
fliN-RT-F	GAAGAGCTGCAGGACGAAAG
fliN-RT-R	GTTCACCACCACCACTTCAC
fliO-RT-F	GTGCTGGGTCTGATTGTTGG
fliO-RT-R	CAATAAACGCTGGCCTTCGA
fliP-RT-F	GAGCTGAAAACTGCGTTCCA
fliP-RT-R	ACCAGCACAAACAGCATCAG
fliQ-RT-F	CATTATGGTGCCTGGGTTGC
fliQ-RT-R	GAAATCCATCAGCTGACCGG
fliR-RT-F	TTTTGTTTCTCGGCTCGGTG
fliR-RT-R	ATATCATAGTGGCGCGGGAA
cheY-RT-F	CAATACCCACGAAGCGGATG
cheY-RT-R	ATCTGTTCACGCTTGGCTTC

cheA-RT-F	GCACTGCTGGAAAATGGTCA
cheA-RT-R	GACAGGCGTGGGTCTAATCT
cheZ-RT-F	CGTCTGCTCGATGATTTGCA
cheZ-RT-R	CTTTCTTCCCGAGCCACATG
cheB-RT-F	TAACGTCAAAGAAGCGGCAG
cheB-RT-R	CAACCGCCAGAACTTTGTCA
cheW-RT-F	CGGGGTTAACGTAATGCAGG
cheW-RT-R	TGTCGGTCACATCACTTGGA
flaM-RT-F	TCTGTGTGGTGAGCGTTGGT
flaM-RT-R	GCGACCGAGGCCAAGATTTG
flgA-RT-F	TGGCAAGTGTATGTACCGGT
flgA-RT-R	CGATCGCCTTTACACACCTG
flgM-RT-F	GCGCTGGATCCTCTGATTGA
flgM-RT-R	TCATCACTTCGCCCAGCAAT
flgN-RT-F	ATCAGTTGCTGCAAACCCTG
flgN-RT-R	GCTAATCCGCGCTTCTTTCA
flgB-RT-F	ACCGAAATGCTGGCCAGTAA
flgB-RT-R	CTGTCGGGCTGATTGGGAAT
flgC-RT-F	GTTCCGCGGATCAAACCTAC
flgC-RT-R	GCAGAAATCATGTCCGCCAT
flgD-RT-F	CGTGATCCGCTTTAACTGGG
flgD-RT-R	CATCGGACTGCAATACCACG
flgE-RT-F	CCGAACGAACAAGGTCTGAC
flgE-RT-R	CAGCTCGATGGTCATGTTGG

flgF-RT-F	CTGATGGTCTGTTTCGCACC
flgF-RT-R	TCATGGCCTTGACCTGCATA
flgG-RT-F	GTCAGCTTTGTAAACCCGCA
flgG-RT-R	GCCCAGCATTTCATCTACCG
flgI-RT-F	GGTTGATGGCACCTCGATTC
flgI-RT-R	TGGCTGACTAACGTTGAGGT
flgJ-RT-F	AGTTTGTGGCGACCTTGTTC
flgJ-RT-R	TTATCGCCATCCCAGCTCTT
flgH-RT-F	GTGCTAGCCCCAATAAACCG
flgH-RT-R	GGTGTTTTCCATCAGCTGCA
flgK-RT-F	GGCTGAGTACACCCAAGTCT
flgK-RT-R	CGCATTTCAAACATCGCACC
flgL-RT-F	CTCAAACAGCAAAGCACCCT
flgL-RT-R	CGGCTTCATCACGGGTATTG
flgO-RT-F	GCACTCTCCGGGCTGTTATT
flgO-RT-R	ATCAGATTGCCCAGCCAGTT
flgP-RT-F	CGGGTGTTAGAGCGTCAACT
flgP-RT-R	AACGAGTTCAGACCGGTGAC
flgT-RT-F	GACAGACCATTCGTTACGCC
flgT-RT-R	TTGCGTCAGATCATTGAGCG
flaC-RT-F	TTGCGATCGACTCATCCCTG
flaC-RT-R	CAGGATCTGCTGCTTGGTCA
flaG-RT-F	AGTCTGCTGCCTTCAACTGA
flaG-RT-R	TTCGCTGAGATCGCTAGTGT

flaH-RT-F	AGAGCCAGCAAACCAAACTG
flaH-RT-R	CCCAGGGCTGTTTTCTGTTC
flaI-RT-F	GAGTTGTTTGCTCCAGAGGC
flaI-RT-R	ACGCACAGCCAATTTATCCC
flaJ-RT-F	GACAGTCAGTTAGATGCGGC
flaJ-RT-R	GGGGTATCACGGGTCAAACT
cheV-RT-F	AGCGCACACAATTAGTCGGA
cheV-RT-R	CAATCGCCAAGCTCATGTCG
cheR-RT-F	AGCCGTTTTCTGGAGTCTCA
cheR-RT-R	TCGCATCAACAACCGACATC
motA-RT-F	TTTTGGCTCTGGATGCGATG
motA-RT-R	TAACCGCCCATGGATTCGTA
motB-RT-F	CGCTCATTGGCTTCATCGAA
motB-RT-R	AATGATCACCTGCTGACCGA
pomA-RT-F	CCAACATGATAGCCAACCCG
pomA-RT-R	GGCAGCCTCGTTCATCAATT
pomB-RT-F	CGTGGGATTTGTCTGCTCAG
pomB-RT-R	GCCAGATTCGGTCGGTTTAC
$fliM_L$-RT-F	AGCGCGAATTTCTGGTTGAG
$fliM_L$-RT-R	ATTGCTGCTGGGTCATTTGG
$fliE_L$-RT-F	AATTTGATGACTGCTGGCGC
$fliE_L$-RT-R	CAGACCGTTAAATGGCAGCG
$fliF_L$-RT-F	CAGTTAGATCGCACCCAACG
$fliF_L$-RT-R	ATTATCACCGGCCACAAACG

fliG$_L$-RT-F	CAGTTCGACATTCTGGAGCG
fliG$_L$-RT-R	CAGGGTCTGCATGATTTCGG
fliH$_L$-RT-F	CAGCAAGTGTTCCATCAGCA
fliH$_L$-RT-R	CAACTGCCACTTGTTCACCA
fliI$_L$-RT-F	AACACCACCGCCGAAATTAC
fliI$_L$-RT-R	TCAACAGCAGCACATCATGG
fliJ$_L$-RT-F	TGGAATTGTTGCAGCAGGTC
fliJ$_L$-RT-R	CTTTTGCAGCAAGGTATCGC
flgB$_L$-RT-F	GCCGAACCGAATTATGTGGC
flgB$_L$-RT-R	TTGCTTGCTTTAACCCGCTG
flgC$_L$-RT-F	GCAAACCGTACGACTCAACA
flgC$_L$-RT-R	TCCCGCGAATGTTCACTTTG
flgD$_L$-RT-F	GCTGAACCTCAACATTGGCA
flgD$_L$-RT-R	ACTTTGATGTTATCCGCCGC
flgE$_L$-RT-F	AGAACAACCCAAGCATGCTG
flgE$_L$-RT-R	AGGTTGCCGGATCGTTGATA
flgF$_L$-RT-F	AACTTCACCACCAACGATGC
flgF$_L$-RT-R	GACCAATTTGAGCTGACCGG
flgG$_L$-RT-F	CGGCACAAGATGTCCAGATG
flgG$_L$-RT-R	TCCTGGGGTCTCATTTTGCT
flgH$_L$-RT-F	CAGCGATGGAAAGGGACAAG
flgH$_L$-RT-R	GGTGTTGCTCTCTTTGCCAA
flgI$_L$-RT-F	GCCAGCACGCATTATCTTCA
flgI$_L$-RT-R	CGTTCACGCTGACATTCGAT

flgJ$_L$-RT-F	CTGGATCGCATCTTTGGTGG
flgJ$_L$-RT-R	CTTGCGCCAACAATACCGAT
flgK$_L$-RT-F	TTGAAAGACCCAGCCGATCT
flgK$_L$-RT-R	GTCTGAATCGCCCAATCACC
flgL$_L$-RT-F	TTGATGATTTGACGGCGGCG
flgL$_L$-RT-R	ACGTGATGCTGCCTCTGGAA
fliC$_L$-RT-F	GTGCCCAAACAACAACAGCA
fliC$_L$-RT-R	CACCCAGCTGTGCCATAGAT
fliD$_L$-RT-F	AAAAGCTTCGTCACCGCCTA
fliD$_L$-RT-R	ACCTGACCTACACTGTTGCG
fliS$_L$-RT-F	TGGATATGGAAAAGGGCGGT
fliS$_L$-RT-R	CATTGCCCAGTCACTTTGCT
lafX-RT-F	AGAAATTGCTTGCCGGACTG
lafX-RT-R	CCTTGTTGCTGTGAGGGTTG
fliK$_L$-RT-F	CAGCACTCCCTCTTACCGAA
fliK$_L$-RT-R	GATGGGGTGTTCATGGCATC
fliL$_L$-RT-F	ACCGCCTGCATTACATGATG
fliL$_L$-RT-R	GCTTTCCCCGGTCATGTTTT
fliA$_L$-RT-F	AACGTGATTTATCCTGCGCC
fliA$_L$-RT-R	TCCAGCCGTCTAACAGTCTG
lafT-RT-F	GATTATCATCGTCGGTGCGG
lafT-RT-R	GCACTGGAGTCGGGATTTTC
lafU-RT-F	GTTCCCAGTCGACTTTGCTG
lafU-RT-R	GCCTTGCAGCTTACCCAAAT

flgA$_L$-RT-F	TAACTTGACCCGGTCCACTC
flgA$_L$-RT-R	TGACCGCACCAGATCTGATT
flgM$_L$-RT-F	CATTTTCAGATCGCGGCTCA
flgM$_L$-RT-R	CACCATGATGCCTACACTGC
flgN$_L$-RT-F	CCGGTTTCAGTAATTGGGTGA
flgN$_L$-RT-R	GATGCAAGCGTTGTTTCGTG
tssB-RT-F	GTGGTCAGCAGGCCGAAATC
tssB-RT-R	TTGGCACCCCTCTTCCAGAC
tssC-RT-F	GGGTATTGCTGCGTTGGTGG
tssC-RT-R	TGGTGGCATGCAAGACTTGG
tssE-RT-F	TGTCATTTCAAGTCACAGCACA
tssE-RT-R	GGTCACCTGCTCGATATTCTCCA
tssF-RT-F	TTGTACGCGGCGATGAAACG
tssF-RT-R	CAAATGACGGTGAGCTGCCC
tssG-RT-F	CAATCGCGCCAAGATGCTGT
tssG-RT-R	ACCTTATCGCCCAGCACCAA
tagH-RT-F	ATCGTGAGCTGGCCAGTCAT
tagH-RT-R	GGCTGGGATGCTGTAGGGAT
tssJ-RT-F	ATGTGGCGATGAACCCGGAT
tssJ-RT-R	CGTAATCGGTGGCCAACAGG
tssK-RT-F	TGCTCGCTTAAGTGCCCTGA
tssK-RT-R	GACCTCACTGACGCCACTGA

tssL-RT-F	ACCATGCCGCACAGCTAGAT
tssL-RT-R	TCTGTCACCACGCGCTGATA
tssH-RT-F	CGAAAGCAAAGCAAACGCCG
tssH-RT-R	CTTCGCCAACCACAATCGGG
vasH-RT-F	ATTCACGACCGCCTGACTGT
vasH-RT-R	GTGATCCAGTGTGCGCTTCG
tagO-RT-F	AGGCTCCTGTGTCGGTTAGC
tagO-RT-R	CCAAATGCGGGTAATGGCGT
tssA-RT-F	TGCGCGAGATGACCTTCAGT
tssA-RT-R	ACTCGCTGCCTAAATCCGCT
tssM-RT-F	CAGTTTGAGTGCGGCGGATC
tssM-RT-R	TCATTGCCAATCACCGCTGC
impA-RT-F	CATTGCAGGGTATGGAGCGC
impA-RT-R	CAAAGGCGAGGCTTGCTCAG
paaR-RT-F	ATTACGGCCAGCCCTGATGT
paaR-RT-R	GCTTATCGTGCGGCTCAAGG
vgrG1-RT-F	GGGCATGCGGTGATTGTGTT
vgrG1-RT-R	CCTGCCCAGCCTGATCTTCA
hcp-RT-F	TCACCTCTGAATCCGTGGGC
hcp-RT-R	GCGCAACGGTGAACTGGAAT
vgrG2-RT-F	CCGGGTTGTGCGTTCAGTTT
vgrG2-RT-R	CACGGTAGCGATTTGTGGGC

$yghF_2$-RT-F	CAAGGATAACGTGCTGGTCG
$yghF_2$-RT-R	AATCCGGTAATTCACGCAGC
$gspD_2$-RT-F	CGCCAGATATCATGCAGTCG
$gspD_2$-RT-R	GCACGCCGAAATTGATGTTG
$gspE_2$-RT-F	GCCTTACTCACTAGCGCCTA
$gspE_2$-RT-R	TGGCGTTGATGAGCTTGATG
$gspF_2$-RT-F	TAGCCGAGCAGTGTGAGAAA
$gspF_2$-RT-R	TGGCGCAAAACAACTCATCA
$gspG_2$-RT-F	AGTGATGGTGGTGATCGTGA
$gspG_2$-RT-R	TCGGGTACACGCTGTTATCC
$gspH_2$-RT-F	CATTAGCTGGTCGACAAGCC
$gspH_2$-RT-R	TACTGTCGAGCCACACTTGT
$gspI_2$-RT-F	GTATTGGCGCAGTAAGTCGG
$gspI_2$-RT-R	TCATAGCGCTGACTCCGTAC
$gspJ_2$-RT-F	TCAGGTGGTTGATGGGGTAC
$gspJ_2$-RT-R	ATTGTAAAAGTCCGTCGCCG
$gspK_2$-RT-F	TTAGATGGGCAAGTCACCGT
$gspK_2$-RT-R	CGAACTGATGGCGTTGAACA
$gspL_2$-RT-F	TTTAGACGTGGCCCAGGATT
$gspL_2$-RT-R	ACATCGGGTTTTGTTTCGGG
$gspM_2$-RT-F	TGTTGGTTGGCGGATTGTAC
$gspM_2$-RT-R	CAATTGCCGTAACAGCTGCT

Primers for EMSAs

fliE-EMSA-F	GCAACGTCTTACCGGGTCTT
fliE-EMSA-R	GCTGTGTGATACATGACGCTGA
fliK-EMSA-F	TTGCTGCGCCGTTTACAAA
fliK-EMSA-R	GAAGTTTGAGACACGGAGCC
flhA-EMSA-F	GCAAGCTGGCATCTCTGTAC
flhA-EMSA-R	CTATCCTCTGTCTACCGCGC
cheY-EMSA-F	AACGGCGGTAAAGTGATTGG
cheY-EMSA-R	CGCTTCTTATACGGCTGACC
flhB-EMSA-F	TTTTGTTTCTCGGCTCGGTG
flhB-EMSA-R	GCGCCTCCTTTACGGTTAC
flaM-EMSA-F	AGCGTGAAATACTCAATATGCCTAT
flaM--EMSA-R	ATGGAGAACTAAGCAAATAGCAGAC
flgO-EMSA-F	CGTAACGTTAGGGTAAAGCGG
flgO-EMSA-R	GTAATGACATTGACACTCCGGC
flgT-EMSA-F	ACCAACTGACGCGCCATC
flgT-EMSA-R	GCCGGAGTGTCAATGTCATT
flgA-EMSA-F	AGCACTTAGACGGATCATCGA
flgA-EMSA-R	AACGTCTCCTGTTATGCAAAA
flgB-EMSA-F	TTTTGTTTCGGTCGCAGGTG
flgB-EMSA-R	ACTTAATGCCAAGACACCGC
flgK-EMSA-F	GGTGGAAGTGATTCAGGTGC

flgK-EMSA-R	TCCTCAATCTGATGTGCCCA
flgL-EMSA-F	CCCAGACCCTGTTCGATGT
flgL-EMSA-R	CGGTAAAACGTCTGGCAAAGA
flaG-EMSA-F	GACATGTCAACTCAAGCCGG
flaG-EMSA-R	GAATTAACCCAGCAGAGACAGC
cheV-EMSA-F	GGTAATAGTTTGCCGTCCCG
cheV-EMSA-R	AACGTGCTACTCCCAGGG
motA-EMSA-F	TCCCCAGGTCTCAAAATCGT
motA-EMSA-R	CATCAAACCTCTGTGCTCGT
pomA-EMSA-F	GCCAATACAATACCACGTTCA
pomA-EMSA-R	CGTATTTGCGTCCCCATAGC
flaC-EMSA-F	ATTTCTTGACATGCCGCGTT
flaC-EMSA-R	ATCTCCGTTAAACTTGCCGC
fliM$_L$-EMSA-F	ATGACGCTGACTCTCCGAAAT
fliM$_L$-EMSA-R	GCTCACTCGGCAACGTCTTA
fliE$_L$-EMSA-F	GCAACGTCTTACCGGGTCTT
fliE$_L$-EMSA-R	GCTGTGTGATACATGACGCTGA
flgB$_L$-EMSA-F	CCTCGCTCAGGCGCTG
flgB$_L$-EMSA-R	GTCCCCCTGCAAACAACG
flgA$_L$-EMSA-F	GACGAGCGATTAATCTGCCG
flgA$_L$-EMSA-R	CGTCTCCTTTAATGCGCAGG
flgK$_L$-EMSA-F	CGGAAGTGATTGACGGACAG
flgK$_L$-EMSA-R	CTCAGTGTAAAAACACCCGACC

flgM$_{L}$-EMSA-F	ACCGATCCACAGGCGCT
flgM$_{L}$-EMSA-R	CCGGCTACTGGCCTAACTTAA
fliC$_{L}$-EMSA-F	GATTGAGCGTCAGCTGCAAG
fliC$_{L}$-EMSA-R	CTTGTTACTGGTATTAGGCGGAA
fliD$_{L}$-EMSA-F	GCAAGCTGGCATCTCTGTAC
fliD$_{L}$-EMSA-R	CTATCCTCTGTCTACCGCGC
Primers for protein cloning	
pMAL-*flaK*-F	CGGGATCCATGCCGGGACTGCAAAAA
pMAL-*flaK*-R	CGGAATTCTCAAACGGCTGCATTTTCC
pMAL-*flaM*-F	CGGGATCCGTGCGACTTAAGCAAAATGAAG
pMAL-*flaM*-R	CGGAATTCTTACTGCGGGATCGCTATC
pMAL-*fliA*-F	CGGGATCCGTGAGTAAAGCCCTGGCTTACA
pMAL-*fliA*-R	CGGAATTCTTATACGGCTGACCAAGCATG
pMAL-*fliA*$_{L}$-F	CGGGATCCATGTGCGGAGCGGCG
pMAL-*fliA*$_{L}$-R	CGGAATTCTTAGTCCGGCTGGTTCCAG

Underlined letters show Kpn1、sacI、Xba1、BamhI、EcoRI or XhoI restriction site.
F/R: The upstream and downstream primers

Table S3 Homologous similarity alignment results of the polar flagellar genes in *P. shigelloides* with *V. cholerae*, *V. parahaemolyticus*, and *A. hydrophila* was blast.

**polar flagella genes (*Plesiomonas shigelloides*)**	***Vibrio cholerae***	***Vibrio parahaemolyticus***	***Aeromonas hydrophila***

	Query Cover	E value	Percent Identity	Query Cover	E value	Percent Identity	Query Cover	E value	Percent Identity
flgT	96%	3.00E-48	30.77%	96%	1.00E-53	30.11%	92%	5.00E-46	30.53%
flgO	77%	2.00E-67	48.80%	77%	6.00E-67	49.30%	54%	3.00E-37	44.00%
flgP	65%	1.00E-29	54.55%	82%	9.00E-32	46.92%	68%	8.00E-19	41.35%
flgN	97%	6.00E-18	34.06%	94%	3.00E-21	36.30%	94%	7.00E-15	32.84%
flgM	63%	2.00E-14	43.28%	55%	7.00E-13	43.10%	66%	5.00E-12	43.06%
flgA	93%	3.00E-35	32.16%	97%	9.00E-34	26.69%	83%	4.00E-30	30.54%
cheV	98%	1.00E-155	67.10%	98%	1.00E-161	69.58%	98%	9.00E-170	73.62%
cheR	100%	1.00E-150	68.71%	100%	2.00E-152	69.78%	98%	2.00E-138	64.23%
flgB	99%	4.00E-54	58.82%	99%	4.00E-53	58.82%	99%	3.00E-49	58.82%
flgC	100%	8.00E-68	71.01%	100%	5.00E-73	73.72%	100%	1.00E-69	71.74%
flgD	72%	3.00E-63	50.00%	67%	6.00E-62	50.99%	72%	1.00E-50	41.82%
flgE	100%	2.00E-143	51.26%	100%	1.00E-150	53.41%	99%	4.00E-132	48.42%
flgF	100%	3.00E-100	57.83%	100%	5.00E-98	55.02%	100%	1.00E-101	56.85%
flgG	100%	5.00E-137	69.47%	100%	1.00E-134	67.94%	99%	2.00E-120	62.45%
flgI	93%	2.00E-179	69.57%	95%	0	71.88%	96%	2.00E-174	66.57%
flgJ	99%	3.00E-81	43.73%	99%	2.00E-89	44.89%	96%	2.00E-67	38.17%
flgH	85%	4.00E-74	55.84%	86%	7.00E-68	52.50%	94%	7.00E-71	51.14%
flgK	99%	8.00E-130	35.20%	99%	7.00E-136	36.93%	99%	1.00E-76	30.67%
flgL	99%	2.00E-70	31.99%	99%	2.00E-66	32.46%	100%	2.00E-28	24.88%
flaC	99%	1.00E-112	51.15%	99%	3.00E-115	51.02%	100%	2.00E-92	46.32%
flaG	96%	3.00E-16	30.26%	46%	2.00E-14	40.62%	54%	3.00E-05	26.51%
flaH	93%	1.00E-37	31.38%	93%	2.00E-36	26.35%	98%	4.00E-63	32.78%
flaI	-	-	-	94%	5.00E-05	31.31%	-	-	-
flaJ	99%	2.00E-58	63.16%	99%	2.00E-58	62.41%	94%	2.00E-20	32.54%
flaK	99%	0	59.68%	99%	0	58.15%	97%	8.00E-162	51.59%
flaM	61%	2.00E-129	60.67%	63%	2.00E-132	58.89%	61%	2.00E-122	57.31%
fliE	80%	1.00E-34	67.09%	100%	8.00E-33	57.14%	84%	2.00E-31	60.24%
fliF	95%	0	53.55%	99%	0	53.49%	95%	0	52.63%
fliG	95%	3.00E-158	69.82%	96%	5.00E-164	70.69%	96%	4.00E-174	74.55%
fliH	80%	2.00E-51	38.17%	80%	7.00E-49	35.77%	80%	2.00E-45	36.36%
fliI	99%	0	77.06%	99%	0	77.12%	99%	0	71.33%
fliJ	95%	2.00E-29	40.29%	95%	7.00E-32	42.45%	78%	5.00E-13	35.09%
fliK	34%	2.00E-27	45.33%	38%	2.00E-23	39.78%	19%	4.00E-18	51.16%
fliL	95%	3.00E-35	36.81%	100%	7.00E-34	35.93%	100%	6.00E-40	41.48%
fliM	95%	0	70.18%	95%	0	71.69%	96%	0	79.10%
fliN	96%	7.00E-61	69.17%	96%	3.00E-62	71.43%	96%	3.00E-59	73.44%
fliO	90%	7.00E-15	36.72%	76%	4.00E-14	33.94%	66%	3.00E-13	40.43%
fliP	85%	1.00E-118	73.42%	86%	2.00E-121	75.56%	86%	2.00E-112	69.91%
fliQ	98%	9.00E-43	68.18%	98%	1.00E-40	65.91%	98%	9.00E-34	57.95%
fliR	98%	7.00E-103	54.62%	98%	1.00E-97	51.15%	99%	3.00E-83	47.15%
flhB	99%	7.00E-169	62.23%	99%	2.00E-169	60.64%	100%	3.00E-136	52.11%
flhA	98%	0	76.91%	98%	0	77.50%	100%	0	71.16%

flhF	97%	9.00E-172	53.77%	97%	2.00E-165	51.61%	99%	3.00E-165	49.07%
flhG	98%	9.00E-145	69.42%	96%	3.00E-146	71.23%	97%	6.00E-142	67.82%
fliA	99%	1.00E-112	63.90%	99%	4.00E-111	63.49%	98%	3.00E-112	69.20%
cheY	96%	6.00E-80	90.16%	96%	5.00E-80	90.16%	99%	6.00E-83	92.06%
cheA	52%	0	77.58%	99%	0	64.37%	100%	0	65.64%
cheZ	100%	4.00E-79	50.62%	100%	6.00E-75	48.99%	100%	4.00E-86	52.70%
cheB	99%	0	68.65%	99%	0	68.62%	99%	0	71.13%
cheW	93%	3.00E-90	80.92%	93%	5.00E-91	83.44%	93%	8.00E-84	76.82%
motA	65%	1.00E-25	32.52%	92%	7.00E-25	27.90%	97%	6.00E-108	61.00%
motB	53%	3.00E-49	52.67%	53%	6.00E-51	50.67%	96%	1.00E-75	45.35%
pomA	90%	2.00E-112	70.74%	98%	2.00E-125	69.08%	97%	1.00E-110	61.29%
pomB	85%	5.00E-106	53.20%	82%	4.00E-105	54.14%	82%	9.00E-100	53.99%

Table S4 Homologous similarity alignment results of the lateral flagellar genes in *P. shigelloides* with *V. cholerae*, *V. parahaemolyticus*, and *A. hydrophila* was blast.

**lateral flagella genes (*Plesiomonas shigelloides*)**	***Vibrio cholerae***			***Vibrio parahaemolyticus***			***Aeromonas hydrophila***		
	**Query Cover**	**E value**	**Percent Identity**	**Query Cover**	**E value**	**Percent Identity**	**Query Cover**	**E value**	**Percent Identity**
**$flhA_L$**	97%	0	50.87%	98%	0	60.58%	99%	0	49.79%
**$flhB_L$**	-	-		-	-	-	-	-	-
**$fliR_L$**	91%	1.00E-43	34.98%	95%	8.00E-86	54.00%	93%	5.00E-58	42.11%
**$fliQ_L$**	86%	5.00E-23	50.65%	91%	7.00E-27	55.56%	91%	5.00E-25	54.32%
**$fliP_L$**	88%	6.00E-93	59.63%	94%	7.00E-119	72.41%	93%	4.00E-90	58.52%
**$fliN_L$**	65%	1.00E-23	46.84%	93%	4.00E-39	56.64%	85%	2.00E-25	42.31%
**$fliM_L$**	96%	6.00E-27	23.49%	93%	4.00E-28	24.14%	98%	4.00E-28	23.18%
**$fliE_L$**	64%	6.00E-15	45.07%	99%	4.00E-28	50.00%	96%	7.00E-17	41.51%
**$fliF_L$**	94%	6.00E-58	29.57%	91%	2.00E-143	42.88%	94%	4.00E-60	30.40%
**$fliG_L$**	96%	2.00E-61	32.92%	98%	2.00E-96	43.38%	94%	4.00E-60	30.40%
**$fliH_L$**	67%	4.00E-17	26.82%	97%	3.00E-54	38.62%	63%	8.00E-16	26.83%
**$fliI_L$**	90%	0	62.19%	98%	0	59.59%	95%	0	62.17%
**$fliJ_L$**	-	-	-	63%	0.007	25.53%	-	-	-
**$flgN_L$**	-	-	-	90%	8.00E-20	38.28%	77%	3.00E-04	30.63%
**$flgM_L$**	-	-	-	51%	1.00E-04	31.51%	-	-	-

flgA$_L$	65%	4.00E-27	35.33%	87%	5.00E-35	34.29%	63%	9.00E-18	32.19%
flgB$_L$	100%	3.00E-20	34.85%	100%	3.00E-44	56.00%	100%	1.00E-32	43.28%
flgC$_L$	97%	8.00E-35	40.15%	97%	5.00E-50	53.19%	97%	6.00E-50	53.28%
flgD$_L$	62%	3.00E-14	25.68%	87%	7.00E-29	32.83%	85%	7.00E-17	28.22%
flgE$_L$	97%	2.00E-53	31.69%	100%	9.00E-101	43.81%	99%	2.00E-54	30.94%
flgF$_L$	99%	2.00E-53	42.74%	99%	5.00E-76	46.67%	99%	4.00E-52	42.91%
flgG$_L$	100%	3.00E-95	55.34%	100%	1.00E-118	68.58%	99%	4.00E-92	52.69%
flgH$_L$	91%	5.00E-43	40.91%	99%	3.00E-85	58.10%	99%	2.00E-54	41.51%
flgI$_L$	99%	3.00E-129	55.78%	99%	7.00E-158	62.79%	99%	4.00E-13<br>1	53.47%
flgJ$_L$	94%	2.00E-61	37.58%	91%	2.00E-62	37.95%	92%	1.00E-61	38.46%
flgK$_L$	52%	2.00E-26	26.61%	58%	2.00E-79	35.01%	49%	8.00E-35	29.15%
flgL$_L$	76%	3.00E-15	27.74%	98%	7.00E-51	35.91%	93%	5.00E-18	32.48%
fliC$_L$	99%	1.00E-51	55.70%	100%	7.00E-83	51.40%	100%	2.00E-79	47.87%
fliD$_L$	94%	1.00E-17	28.81%	94%	2.00E-45	26.74%	98%	2.00E-41	27.10%
fliS$_L$	94%	6.00E-28	35.48%	92%	4.00E-36	45.45%	93%	8.00E-23	35.77%
lafX	46%	0.036	44.44%	-	-	-	-	-	-
fliK$_L$	19%	1.00E-13	43.04%	32%	6.00E-22	37.31%	15%	2.00E-08	42.19%
fliL$_L$	-	-	-	70%	6.00E-12	30.36%	98%	9.00E-11	27.27%
fliA$_L$	95%	5.00E-41	33.47%	87%	2.00E-57	45.87%	87%	9.00E-39	35.37%
lafT	64%	5.00E-04	22.63%	100%	5.00E-99	52.28%	87%	2.00E-05	23.74%
lafU	57%	2.00E-14	23.89%	65%	1.00E-65	38.87%	58%	5.00E-10	24.21%

Table S5 Transcriptome revealed differential expressed genes related to FlaK of the *P. shigelloides*

Gene id	Gene name	log2FoldChange	pvalue
GBN32_RS01260	*GBN32_RS01260*	-1.721176547	0.000477659
GBN32_RS02175	*purD*	-1.839791615	6.30E-05
GBN32_RS03360	*flgT*	-3.450079404	3.51E-13
GBN32_RS03365	*flgO*	-4.434691216	7.95E-19
GBN32_RS03370	*flgP*	-4.620606083	9.45E-15
GBN32_RS03375	*flgN*	-3.169798881	8.44E-12
GBN32_RS03380	*flgM*	-3.060507779	2.01E-11
GBN32_RS03390	*cheV*	-1.683550738	7.65E-05
GBN32_RS03395	*cheR*	-1.36877948	0.001267733
GBN32_RS03400	*flgB*	-5.314214959	1.59E-18
GBN32_RS03405	*flgC*	-4.510606049	2.74E-16
GBN32_RS03410	*flgD*	-3.051162993	4.54E-10
GBN32_RS03415	*flgE*	-3.258114021	1.94E-12
GBN32_RS03420	*GBN32_RS03420*	-5.36632565	2.18E-21
GBN32_RS03425	*flgF*	-5.764623476	4.03E-24
GBN32_RS03430	*flgG*	-5.339987708	6.07E-24
GBN32_RS03435	*flgI*	-4.565864554	1.16E-17
GBN32_RS03440	*flgJ*	-4.709370957	3.84E-18
GBN32_RS03445	*flgH*	-5.338680592	9.24E-20
GBN32_RS03450	*flgK*	-4.395765105	1.13E-19
GBN32_RS03455	*flgL*	-3.593804488	1.24E-14
GBN32_RS03460	*GBN32_RS03460*	-3.110760348	1.14E-08
GBN32_RS03515	*GBN32_RS03515*	-5.706461177	5.76E-29
GBN32_RS03520	*GBN32_RS03520*	-6.939700114	0.000315208
GBN32_RS03525	*fliD*	-4.553169301	9.54E-11
GBN32_RS03540	*GBN32_RS03540*	-1.38089946	0.001193552
GBN32_RS03550	*fliE*	-3.115898658	1.74E-11
GBN32_RS03555	*fliF*	-3.632094693	9.36E-15
GBN32_RS03560	*fliG*	-3.132331622	2.42E-11
GBN32_RS03565	*fliH*	-3.360318433	8.77E-13
GBN32_RS03570	*fliI*	-3.066180187	3.31E-11
GBN32_RS03575	*fliJ*	-2.636095781	2.42E-08
GBN32_RS03585	*fliK*	-3.554807901	2.46E-13
GBN32_RS03590	*fliL*	-4.243304771	8.09E-15
GBN32_RS03595	*fliM*	-3.276752731	7.46E-12
GBN32_RS03600	*fliN*	-3.389891368	1.66E-12
GBN32_RS03605	*fliO*	-3.675748822	2.65E-12

GBN32_RS03610	*fliP*	-3.250688874	4.37E-09
GBN32_RS03615	*fliQ*	-2.376274379	0.000126706
GBN32_RS03620	*fliR*	-1.539222125	0.001672817
GBN32_RS03630	*flhA*	-4.283137235	6.88E-18
GBN32_RS03635	*flhF*	-4.725637587	1.72E-20
GBN32_RS03640	*flhG*	-4.348519533	3.85E-18
GBN32_RS03645	*fliA*	-3.71062637	3.61E-14
GBN32_RS03650	*GBN32_RS03650*	-3.37698035	4.59E-13
GBN32_RS03655	*cheY*	-3.582360231	1.16E-14
GBN32_RS03660	*cheZ*	-3.650718639	3.23E-15
GBN32_RS03665	*cheA*	-3.167389751	6.43E-12
GBN32_RS03670	*cheB*	-3.723530621	3.84E-14
GBN32_RS03675	*cheW*	-3.54794557	4.19E-13
GBN32_RS03680	*GBN32_RS03680*	-2.091482382	1.31E-05
GBN32_RS03780	*GBN32_RS03780*	-5.374897341	5.73E-22
GBN32_RS03945	*GBN32_RS03945*	-1.321944537	0.00183577
GBN32_RS04020	*purE*	-1.586079682	0.001268593
GBN32_RS04150	*GBN32_RS04150*	-5.51371938	5.90E-27
GBN32_RS04155	*GBN32_RS04155*	-5.141673124	4.96E-16
GBN32_RS04200	*GBN32_RS04200*	-4.126727884	0.000376589
GBN32_RS04310	*GBN32_RS04310*	-5.352244031	7.93E-25
GBN32_RS04720	*napF*	-1.585247892	0.000956767
GBN32_RS04730	*napA*	-1.500568844	0.00056685
GBN32_RS04735	*napG*	-1.691855736	0.000196967
GBN32_RS04740	*napH*	-1.550655844	0.001323137
GBN32_RS04745	*napB*	-1.719158977	0.00025933
GBN32_RS05100	*GBN32_RS05100*	-2.181292734	3.15E-05
GBN32_RS06970	*GBN32_RS06970*	-4.103540192	3.72E-12
GBN32_RS07080	*panB*	-1.380628619	0.001512739
GBN32_RS07285	*pyrI*	-1.899816662	2.04E-05
GBN32_RS07290	*pyrB*	-1.375751988	0.001515884
GBN32_RS08155	*pomA*	-3.531635701	3.92E-11
GBN32_RS08160	*GBN32_RS08160*	-1.640336016	0.000311328
GBN32_RS08360	*nadD*	-1.539222125	0.001672817
GBN32_RS09075	*GBN32_RS09075*	-2.040088919	8.70E-06
GBN32_RS09390	*GBN32_RS09390*	-3.080056346	6.96E-11
GBN32_RS09805	*GBN32_RS09805*	-2.115062195	9.89E-05
GBN32_RS10050	*GBN32_RS10050*	-1.580034173	0.001745102
GBN32_RS10460	*tssF*	-1.294346086	0.002271356
GBN32_RS10470	*tagH*	-1.546113901	0.000253072
GBN32_RS10475	*tssJ*	-1.458190191	0.000839501
GBN32_RS10480	*tssK*	-1.70569483	6.11E-05
GBN32_RS10485	*icmH*	-2.093772591	1.91E-06
GBN32_RS10490	*tssH*	-1.845714899	1.41E-05

GBN32_RS10495	*GBN32_RS10495*	-1.762045184	3.81E-05
GBN32_RS10500	*tagO*	-1.796353954	2.73E-05
GBN32_RS10505	*tssA*	-2.097062587	1.20E-06
GBN32_RS10510	*tssM*	-1.909456208	7.43E-06
GBN32_RS10515	*GBN32_RS10515*	-1.731316022	4.57E-05
GBN32_RS10520	*GBN32_RS10520*	-1.765857084	5.09E-05
GBN32_RS10525	*GBN32_RS10525*	-2.070971986	1.54E-06
GBN32_RS11280	*metF*	-2.221381847	7.57E-05
GBN32_RS11650	*GBN32_RS11650*	-3.846803942	1.34E-15
GBN32_RS11740	*glnL*	-1.721385881	0.000651011
GBN32_RS12155	*GBN32_RS12155*	-2.782319525	5.01E-09
GBN32_RS12630	*GBN32_RS12630*	-2.266776921	5.75E-07
GBN32_RS12635	*lafU*	-3.295947018	1.27E-11
GBN32_RS12640	*lafT*	-3.886494412	2.88E-14
GBN32_RS12645	$fliK_L$	-3.052502541	7.19E-10
GBN32_RS12650	$fliL_L$	-5.212752847	1.15E-21
GBN32_RS12655	*lafX*	-3.969549652	1.99E-11
GBN32_RS12660	$fliS_L$	-4.038181339	1.86E-14
GBN32_RS12665	$fliD_L$	-4.701742833	2.04E-18
GBN32_RS12670	$fliC_L$	-6.427506541	5.65E-31
GBN32_RS12675	*GBN32_RS12675*	-2.028382214	6.04E-06
GBN32_RS12680	$flgL_L$	-2.282395774	3.22E-05
GBN32_RS12685	$flgK_L$	-2.685827698	8.79E-09
GBN32_RS12690	$flgJ_L$	-2.93279596	1.82E-06
GBN32_RS12700	$flgH_L$	-2.41062157	3.40E-06
GBN32_RS12705	$flgG_L$	-2.611528628	5.16E-07
GBN32_RS12710	$flgF_L$	-2.686454041	5.32E-07
GBN32_RS12715	$flgE_L$	-2.269060298	5.30E-06
GBN32_RS12725	$flgC_L$	-3.821013226	7.93E-09
GBN32_RS12730	$flgB_L$	-4.905061863	1.20E-12
GBN32_RS12745	$flgM_L$	-1.756046885	9.40E-05
GBN32_RS12750	$flgN_L$	-4.221065425	1.89E-11
GBN32_RS12755	$fliI_L$	-2.401672957	4.16E-06
GBN32_RS12760	$fliH_L$	-3.817026658	6.20E-12
GBN32_RS12765	$fliG_L$	-2.809453	7.31E-08
GBN32_RS12770	$fliF_L$	-2.507041015	2.59E-07
GBN32_RS12775	$fliE_L$	-3.955392116	5.57E-07
GBN32_RS12780	$fliM_L$	-5.398414574	7.04E-14
GBN32_RS12785	$fliN_L$	-9.736946449	2.65E-15
GBN32_RS12790	$fliP_L$	-2.876706243	5.26E-06
GBN32_RS12795	$fliQ_L$	-8.026552651	2.40E-07
GBN32_RS12800	$fliR_L$	-3.298535226	4.91E-07
GBN32_RS12825	*glmU*	-2.155495659	5.13E-07
GBN32_RS13100	*wecC*	-1.389752861	0.001148498

GBN32_RS13110	*GBN32_RS13110*	-1.369198983	0.001213538
GBN32_RS13115	*wecA*	-1.588637303	0.000215142
GBN32_RS13360	*GBN32_RS13360*	-1.778750624	4.67E-05
GBN32_RS13365	*GBN32_RS13365*	-3.567011091	2.73E-13
GBN32_RS13940	*GBN32_RS13940*	-4.126727884	0.000376589
GBN32_RS14350	*GBN32_RS14350*	-6.139489819	2.07E-21
GBN32_RS14865	*GBN32_RS14865*	-3.531378822	2.31E-05
GBN32_RS15365	*pspF*	-1.362743659	0.002219762
GBN32_RS15535	*GBN32_RS15535*	-2.904884487	2.39E-10
GBN32_RS16275	*GBN32_RS16275*	-1.839753805	2.48E-05
GBN32_RS17170	*GBN32_RS17170*	-2.389256155	1.23E-07
GBN32_RS17735	*GBN32_RS17735*	-1.653387716	0.000193766
GBN32_RS17785	*GBN32_RS17785*	-2.461058124	0.000519431
GBN32_RS18150	*GBN32_RS18150*	-1.741601376	0.000619351
GBN32_RS01685	*GBN32_RS01685*	1.565761121	0.000256933
GBN32_RS01690	*prpC*	1.815151087	2.33E-05
GBN32_RS01845	*GBN32_RS01845*	2.589342445	0.000151266
GBN32_RS03685	*GBN32_RS03685*	1.743643184	5.65E-05
GBN32_RS03710	*GBN32_RS03710*	2.448637175	2.57E-07
GBN32_RS03730	*iolC*	2.342725017	1.09E-07
GBN32_RS03735	*iolE*	2.914802162	3.89E-11
GBN32_RS03740	*iolB*	2.076588715	2.93E-06
GBN32_RS04060	*GBN32_RS04060*	1.668700412	0.002227069
GBN32_RS05295	*GBN32_RS05295*	1.51539183	0.000547892
GBN32_RS14895	*GBN32_RS14895*	1.344455546	0.001395862
GBN32_RS17240	*GBN32_RS17240*	1.422073169	0.000861632
GBN32_RS17900	*GBN32_RS17900*	1.978109246	3.40E-06

Table S6 Transcriptome revealed differential expressed genes related to FlaM of the *P. shigelloides*

Gene id	Gene name	log2FoldChange	pvalue
GBN32_RS18160	*GBN32_RS18160*	-1.184936985	0.008971824
GBN32_RS18150	*GBN32_RS18150*	-3.031837048	5.36E-05
GBN32_RS18145	*GBN32_RS18145*	-9.745091788	2.22E-15
GBN32_RS18140	*GBN32_RS18140*	-11.21114432	1.85E-24
GBN32_RS18125	*GBN32_RS18125*	-3.674624061	0.003346055
GBN32_RS18090	*thiE*	-2.311580845	0.020952111
GBN32_RS18005	*GBN32_RS18005*	-1.091209253	0.036410837

GBN32_RS17945	*GBN32_RS17945*	-1.635021061	0.044489089
GBN32_RS17785	*GBN32_RS17785*	-6.092286088	0.012730848
GBN32_RS17780	*GBN32_RS17780*	-2.040369469	0.002205393
GBN32_RS17730	*GBN32_RS17730*	-1.461679882	0.000955263
GBN32_RS17555	*GBN32_RS17555*	-1.217336708	0.003802828
GBN32_RS17310	*GBN32_RS17310*	-2.109885324	0.01323105
GBN32_RS17300	*GBN32_RS17300*	-2.760693275	0.011832427
GBN32_RS17295	*GBN32_RS17295*	-1.218398957	0.032118678
GBN32_RS17205	*nuoH*	-1.126815117	0.007126204
GBN32_RS17190	*nuoK*	-1.048184153	0.020506905
GBN32_RS17105	*GBN32_RS17105*	-1.695648949	0.002227069
GBN32_RS17035	*GBN32_RS17035*	-2.102837809	0.023070854
GBN32_RS16075	*purL*	-1.130071157	0.006855185
GBN32_RS15930	*suhB*	-1.24264247	0.005370407
GBN32_RS15890	*aceA*	-1.17742048	0.004637467
GBN32_RS15885	*aceB*	-1.035779046	0.012620773
GBN32_RS15855	*GBN32_RS15855*	-1.024950206	0.041996547
GBN32_RS15535	*GBN32_RS15535*	-4.18841884	1.47E-17
GBN32_RS15525	*GBN32_RS15525*	-1.736900922	0.001181415
GBN32_RS15520	*GBN32_RS15520*	-1.102233845	0.016909258
GBN32_RS15430	*GBN32_RS15430*	-1.406761227	0.011094044
GBN32_RS14875	*thrC*	-1.845028902	2.35E-05
GBN32_RS14865	*GBN32_RS14865*	-1.301561057	0.031520396
GBN32_RS14860	*thrA*	-1.291434406	0.002838844
GBN32_RS14815	*gltB*	-1.438233783	0.000597764
GBN32_RS14810	*GBN32_RS14810*	-1.924051415	6.23E-06
GBN32_RS14790	*GBN32_RS14790*	-1.864226111	0.002617946
GBN32_RS14745	*leuA*	-1.362007094	0.00153109
GBN32_RS14740	*leuB*	-1.954846601	9.04E-06
GBN32_RS14735	*leuC*	-1.918836718	8.04E-06
GBN32_RS14730	*leuD*	-1.532566424	0.000365963
GBN32_RS14350	*GBN32_RS14350*	-5.072114195	7.62E-10
GBN32_RS14305	*mutL*	-1.313767793	0.002340985
GBN32_RS14175	*groL*	-1.289080054	0.002007799
GBN32_RS14035	*pntB*	-1.238144549	0.003581694
GBN32_RS13920	*GBN32_RS13920*	-1.761339381	0.009730816
GBN32_RS13510	*moaC*	-1.027263995	0.018435175
GBN32_RS13495	*modA*	-1.092138818	0.013305171
GBN32_RS13365	*GBN32_RS13365*	-1.092724945	0.021834404
GBN32_RS13225	*thiC*	-1.415080235	0.043692991
GBN32_RS13215	*GBN32_RS13215*	-6.259859697	0.007243414
GBN32_RS13145	*GBN32_RS13145*	-1.008838812	0.036223839
GBN32_RS12705	*flgG*	-1.452027165	0.018121122
GBN32_RS12670	*GBN32_RS12670*	-2.434461355	1.32E-05

GBN32_RS12660	*fliS*	-1.473321271	0.021872151
GBN32_RS12635	*motA*	-1.858031112	0.001563952
GBN32_RS12600	*rbsD*	-1.483079802	0.010235822
GBN32_RS12595	*rbsA*	-1.005360682	0.023142029
GBN32_RS12580	*GBN32_RS12580*	-1.325884648	0.021979843
GBN32_RS12575	*GBN32_RS12575*	-1.16403253	0.042612006
GBN32_RS12570	*GBN32_RS12570*	-1.372918575	0.005822763
GBN32_RS12560	*bcsZ*	-1.325180589	0.002731747
GBN32_RS12555	*bcsB*	-1.049779827	0.011945237
GBN32_RS12505	*yegD*	-1.790088943	0.045435959
GBN32_RS12435	*ilvG*	-2.28687075	2.55E-07
GBN32_RS12430	*ilvM*	-2.671165642	1.14E-07
GBN32_RS12425	*GBN32_RS12425*	-3.128319478	6.77E-12
GBN32_RS12420	*ilvD*	-2.595620168	5.64E-09
GBN32_RS12415	*ilvA*	-2.378211838	6.24E-08
GBN32_RS12410	*GBN32_RS12410*	-2.06576647	0.006891763
GBN32_RS12405	*ilvY*	-1.203691363	0.040567706
GBN32_RS12385	*zupT*	-4.027036642	5.35E-14
GBN32_RS12180	*GBN32_RS12180*	-1.011450249	0.017177367
GBN32_RS12175	*GBN32_RS12175*	-1.108547288	0.016962395
GBN32_RS12120	*glpC*	-3.92820113	4.70E-15
GBN32_RS12115	*glpB*	-4.166003128	1.46E-13
GBN32_RS12105	*glpA*	-5.594616856	3.29E-19
GBN32_RS12100	*glpT*	-3.390237569	1.10E-11
GBN32_RS12095	*glpQ*	-2.102159978	7.66E-06
GBN32_RS12080	*pldB*	-1.353266578	0.003981114
GBN32_RS12065	*asd*	-1.516405824	0.000336138
GBN32_RS11910	*GBN32_RS11910*	-1.454651526	0.001131195
GBN32_RS11905	*dapF*	-1.011605561	0.019433496
GBN32_RS11900	*GBN32_RS11900*	-1.834398202	4.20E-05
GBN32_RS11890	*yigB*	-1.374883107	0.006826807
GBN32_RS11810	*fabG*	-1.417300708	0.010901385
GBN32_RS11700	*fabY*	-1.562994041	0.000658314
GBN32_RS11695	*GBN32_RS11695*	-1.052619737	0.013654531
GBN32_RS11680	*recG*	-1.42336714	0.002489392
GBN32_RS11675	*trmH*	-1.009441963	0.02005755
GBN32_RS11650	*GBN32_RS11650*	-2.091028672	5.63E-06
GBN32_RS11465	*GBN32_RS11465*	-1.555176558	0.001927172
GBN32_RS11430	*cysE*	-1.548159682	0.000739197
GBN32_RS11420	*cpxA*	-1.090506295	0.022090381
GBN32_RS11360	*glpK*	-1.60630963	0.000165577
GBN32_RS11315	*priA*	-1.512656234	0.001615132
GBN32_RS11285	*GBN32_RS11285*	-1.273707592	0.00293686
GBN32_RS11280	*metF*	-1.827335256	0.001005496

GBN32_RS11270	*ppc*	-1.372431501	0.001247412
GBN32_RS11255	*argB*	-1.933607368	8.30E-06
GBN32_RS11245	*argH*	-1.063275541	0.011094357
GBN32_RS11030	*rplP*	-1.07490936	0.010008764
GBN32_RS11020	*rplV*	-1.191885136	0.004539184
GBN32_RS11015	*rpsS*	-1.004410879	0.016545451
GBN32_RS11010	*rplB*	-1.081858431	0.009355834
GBN32_RS11005	*rplW*	-1.079233787	0.010591016
GBN32_RS11000	*rplD*	-1.323818184	0.001613947
GBN32_RS10990	*rpsJ*	-1.117848214	0.008015289
GBN32_RS10985	*GBN32_RS10985*	-1.662054586	0.002204669
GBN32_RS10715	*GBN32_RS10715*	-1.355895891	0.001551863
GBN32_RS10710	*glsA*	-1.219060928	0.004546464
GBN32_RS10700	*GBN32_RS10700*	-1.479372287	0.007401946
GBN32_RS10695	*GBN32_RS10695*	-1.367434509	0.032364314
GBN32_RS10605	*GBN32_RS10605*	-2.087731817	0.001019268
GBN32_RS10525	*GBN32_RS10525*	-1.816281525	2.70E-05
GBN32_RS10520	*GBN32_RS10520*	-2.512734905	6.57E-08
GBN32_RS10515	*GBN32_RS10515*	-1.920983679	9.21E-06
GBN32_RS10510	*tssM*	-2.033679151	2.24E-06
GBN32_RS10505	*tssA*	-1.999436232	5.01E-06
GBN32_RS10500	*tagO*	-2.325673992	1.50E-07
GBN32_RS10495	*GBN32_RS10495*	-1.976772248	5.68E-06
GBN32_RS10490	*tssH*	-2.041074858	2.08E-06
GBN32_RS10485	*icmH*	-1.882964311	4.24E-05
GBN32_RS10480	*tssK*	-1.786650092	3.40E-05
GBN32_RS10475	*tssJ*	-1.549557098	0.000809139
GBN32_RS10470	*tagH*	-1.851588696	1.66E-05
GBN32_RS10465	*tssG*	-1.479124221	0.000532109
GBN32_RS10460	*tssF*	-1.037887952	0.013607367
GBN32_RS10440	*GBN32_RS10440*	-1.344317154	0.001327122
GBN32_RS10410	*GBN32_RS10410*	-1.143427092	0.006808014
GBN32_RS09965	*mtr*	-1.035810051	0.018886693
GBN32_RS09505	*menE*	-1.210356315	0.006637671
GBN32_RS09305	*htpG*	-1.013988725	0.014699592
GBN32_RS08855	*hisD*	-1.356240647	0.014532818
GBN32_RS08850	*hisC*	-1.445369647	0.003519041
GBN32_RS08815	*GBN32_RS08815*	-1.156317984	0.005453493
GBN32_RS08810	*artQ*	-1.63317908	0.00010926
GBN32_RS08805	*artM*	-1.408705189	0.000906466
GBN32_RS08795	*rlmC*	-1.311050447	0.025864487
GBN32_RS08790	*GBN32_RS08790*	-1.358506483	0.001762692
GBN32_RS08595	*odhB*	-1.108150762	0.007625483
GBN32_RS08580	*sdhA*	-1.069420403	0.010101523

GBN32_RS08560	*phbB*	-1.095563974	0.008574771
GBN32_RS08340	*mrdB*	-1.093307298	0.017797981
GBN32_RS08310	*GBN32_RS08310*	-1.227238136	0.010967701
GBN32_RS08300	*GBN32_RS08300*	-1.685112041	0.004472386
GBN32_RS08095	*clpB*	-1.059045141	0.01074898
GBN32_RS08035	*GBN32_RS08035*	-1.429681157	0.001233752
GBN32_RS07660	*GBN32_RS07660*	-1.126834391	0.017000786
GBN32_RS07480	*recD*	-1.193620467	0.008409002
GBN32_RS07475	*GBN32_RS07475*	-1.327230152	0.014956827
GBN32_RS07200	*GBN32_RS07200*	-1.068776918	0.020589948
GBN32_RS07115	*hrpB*	-1.353383401	0.02655207
GBN32_RS07075	*panC*	-1.085702162	0.016802927
GBN32_RS07025	*GBN32_RS07025*	-1.158485235	0.010519844
GBN32_RS07020	*cueO*	-1.022108114	0.026962251
GBN32_RS07010	*acnB*	-1.218447468	0.003457942
GBN32_RS06960	*aceF*	-1.576826786	0.000222822
GBN32_RS06955	*aceE*	-1.623621476	0.000140801
GBN32_RS06600	*tldD*	-1.058203352	0.012791528
GBN32_RS06260	*cadB*	-2.750319654	8.88E-10
GBN32_RS05630	*hcp1*	-1.427016878	0.00392515
GBN32_RS05615	*putP*	-1.088918896	0.008986497
GBN32_RS05350	*GBN32_RS05350*	-1.247037521	0.004107256
GBN32_RS05345	*GBN32_RS05345*	-1.541210063	0.000338391
GBN32_RS05340	*GBN32_RS05340*	-1.768669823	0.001896709
GBN32_RS05330	*GBN32_RS05330*	-1.504267423	0.000385286
GBN32_RS05190	*gspL*	-1.606904322	0.000371717
GBN32_RS05185	*gspK*	-1.356628417	0.003394775
GBN32_RS05180	*gspJ*	-1.739291626	0.00168007
GBN32_RS05175	*gspI*	-2.185298105	0.000339042
GBN32_RS05170	*gspH*	-1.340418821	0.0037935
GBN32_RS05165	*gspG*	-1.635060037	0.000538104
GBN32_RS05160	*gspF*	-1.203016575	0.009143534
GBN32_RS05155	*gspE*	-1.619291792	0.000217192
GBN32_RS05150	*gspD*	-1.311593619	0.002436294
GBN32_RS05145	*gspC*	-1.223852121	0.013452294
GBN32_RS05045	*glpD*	-2.517219714	2.27E-08
GBN32_RS05015	*livG*	-1.22743507	0.013338011
GBN32_RS04890	*ruvC*	-1.118272343	0.007859991
GBN32_RS03825	*mepA*	-1.212059927	0.006503888
GBN32_RS03695	*iolD*	-1.462197431	0.001229473
GBN32_RS03635	*flhF*	-1.249560746	0.02851012
GBN32_RS03455	*flgL*	-2.920152988	1.81E-10
GBN32_RS03450	*flgK*	-2.77988521	2.54E-10
GBN32_RS03445	*flgJ*	-1.049327517	0.019176968

GBN32_RS03440	*flgI*	-1.242282139	0.003946322
GBN32_RS03435	*flgH*	-1.201394416	0.021235214
GBN32_RS03430	*flgG*	-1.38850423	0.018748976
GBN32_RS03425	*flgF*	-1.934411651	0.001726885
GBN32_RS03415	*flgE*	-2.720194755	1.59E-08
GBN32_RS03410	*flgD*	-2.597215225	2.46E-07
GBN32_RS03405	*flgC*	-1.619822223	0.001635033
GBN32_RS03400	*flgB*	-1.023468024	0.027410684
GBN32_RS03385	*flgA*	-1.509182449	0.000602599
GBN32_RS03380	*flgM*	-1.274339486	0.003352144
GBN32_RS03375	*flgN*	-1.016433452	0.01418848
GBN32_RS03370	*flgP*	-2.742854643	0.001197455
GBN32_RS03365	*flgO*	-2.105056146	6.64E-05
GBN32_RS03360	*flgT*	-1.319384023	0.001587347
GBN32_RS02950	*bioF*	-1.137657627	0.012011792
GBN32_RS02385	*putA*	-1.42863415	0.000647779
GBN32_RS02290	*metH*	-1.045109974	0.015669942
GBN32_RS02285	*lysC*	-1.75167629	0.000110756
GBN32_RS02220	*rpoC*	-1.376925446	0.000983637
GBN32_RS02175	*purD*	-1.22772113	0.004921831
GBN32_RS01100	*GBN32_RS01100*	-1.527743566	0.000612885
GBN32_RS00760	*GBN32_RS00760*	-1.077757735	0.029477516
GBN32_RS00445	*GBN32_RS00445*	-1.779336564	3.37E-05
GBN32_RS18205	*GBN32_RS18205*	1.092111297	0.01495922
GBN32_RS18000	*GBN32_RS18000*	1.17699449	0.007527084
GBN32_RS17925	*GBN32_RS17925*	1.779821097	0.019137366
GBN32_RS17680	*GBN32_RS17680*	1.561442347	0.034088382
GBN32_RS17650	*pstB*	1.122030894	0.0109791
GBN32_RS17260	*fabA*	1.462244917	0.000544076
GBN32_RS17250	*matP*	1.415160284	0.001709694
GBN32_RS17135	*GBN32_RS17135*	1.4826463	0.033173684
GBN32_RS17045	*GBN32_RS17045*	1.091491897	0.014046511
GBN32_RS16860	*GBN32_RS16860*	1.055104437	0.021321039
GBN32_RS16490	*GBN32_RS16490*	1.093109829	0.015450737
GBN32_RS16375	*GBN32_RS16375*	1.134414722	0.00958207
GBN32_RS16315	*GBN32_RS16315*	1.004580413	0.021277907
GBN32_RS16260	*GBN32_RS16260*	1.5625039	0.000384796
GBN32_RS16250	*GBN32_RS16250*	1.227145228	0.008774059
GBN32_RS16065	*GBN32_RS16065*	1.046331282	0.012590401
GBN32_RS15945	*GBN32_RS15945*	1.417278834	0.000749273
GBN32_RS15940	*iscR*	1.556911777	0.000237896
GBN32_RS15315	*GBN32_RS15315*	1.899461532	0.010811714
GBN32_RS15235	*GBN32_RS15235*	3.710916785	0.002133716
GBN32_RS15130	*GBN32_RS15130*	1.405858194	0.000836965

GBN32_RS14840	*GBN32_RS14840*	1.146920193	0.010231927
GBN32_RS14725	*GBN32_RS14725*	1.126699157	0.011743974
GBN32_RS14720	*GBN32_RS14720*	1.438397577	0.000854279
GBN32_RS14185	*GBN32_RS14185*	1.061207696	0.030316257
GBN32_RS14135	*GBN32_RS14135*	1.4080923	0.004047714
GBN32_RS14075	*GBN32_RS14075*	5.955778185	0.012730848
GBN32_RS13800	*GBN32_RS13800*	1.253602739	0.002762761
GBN32_RS13340	*GBN32_RS13340*	5.54846788	0.040964585
GBN32_RS13315	*GBN32_RS13315*	1.265655975	0.009650452
GBN32_RS12820	*GBN32_RS12820*	1.284793248	0.002299383
GBN32_RS12615	*GBN32_RS12615*	1.099596884	0.037792586
GBN32_RS11635	*GBN32_RS11635*	1.615377058	0.017539069
GBN32_RS11605	*GBN32_RS11605*	2.232042056	0.020952111
GBN32_RS11475	*GBN32_RS11475*	1.010207763	0.026363892
GBN32_RS10885	*GBN32_RS10885*	1.098287537	0.03287854
GBN32_RS10875	*ydfZ*	1.844475613	1.45E-05
GBN32_RS10810	*GBN32_RS10810*	1.665262515	0.000940937
GBN32_RS10805	*GBN32_RS10805*	1.112752882	0.026598839
GBN32_RS10750	*GBN32_RS10750*	1.370928389	0.014399462
GBN32_RS10620	*GBN32_RS10620*	1.681082781	0.019987884
GBN32_RS10550	*GBN32_RS10550*	1.467904096	0.011696955
GBN32_RS10380	*nfsB*	1.120956801	0.008466775
GBN32_RS10345	*GBN32_RS10345*	1.040021396	0.015419617
GBN32_RS10025	*rmf*	1.187704955	0.00428636
GBN32_RS09895	*GBN32_RS09895*	1.376528776	0.00314182
GBN32_RS09710	*GBN32_RS09710*	1.015058884	0.018868234
GBN32_RS09690	*GBN32_RS09690*	1.201587994	0.024220592
GBN32_RS09665	*GBN32_RS09665*	1.551130816	0.004739774
GBN32_RS09660	*msrP*	1.112615203	0.009932617
GBN32_RS09620	*pyrF*	1.22324041	0.021473972
GBN32_RS09510	*GBN32_RS09510*	1.081300847	0.015022627
GBN32_RS09410	*yfcD*	1.222987517	0.034237201
GBN32_RS09405	*yfcE*	1.073089288	0.013637427
GBN32_RS09395	*GBN32_RS09395*	1.368440224	0.031520396
GBN32_RS09060	*GBN32_RS09060*	1.973496066	0.01323105
GBN32_RS08905	*GBN32_RS08905*	1.25160522	0.010967701
GBN32_RS08735	*GBN32_RS08735*	1.138933725	0.007472071
GBN32_RS08730	*GBN32_RS08730*	2.451063379	3.75E-06
GBN32_RS08540	*GBN32_RS08540*	1.731626649	0.000137376
GBN32_RS08195	*GBN32_RS08195*	1.105464641	0.046223025
GBN32_RS08150	*xseB*	1.042479046	0.018415512
GBN32_RS08090	*GBN32_RS08090*	1.537841858	0.01296303
GBN32_RS07940	*GBN32_RS07940*	1.1273954	0.021281883
GBN32_RS07930	*GBN32_RS07930*	1.13277134	0.013483701

GBN32_RS07735	*GBN32_RS07735*	1.379263197	0.001627547
GBN32_RS07685	*GBN32_RS07685*	1.086095072	0.011594271
GBN32_RS07680	*zapA*	1.026829384	0.014837238
GBN32_RS07345	*GBN32_RS07345*	1.131528948	0.009914968
GBN32_RS07065	*GBN32_RS07065*	1.108713018	0.011438103
GBN32_RS06345	*GBN32_RS06345*	1.097062198	0.01485635
GBN32_RS06335	*GBN32_RS06335*	1.016983863	0.019845673
GBN32_RS06010	*trmB*	1.237402974	0.013213222
GBN32_RS05935	*hdcC*	2.31881554	0.014485426
GBN32_RS05900	*speF*	1.505374906	0.000356125
GBN32_RS05870	*GBN32_RS05870*	1.583472477	0.00058808
GBN32_RS04965	*GBN32_RS04965*	1.011990768	0.030870438
GBN32_RS04920	*GBN32_RS04920*	1.087159969	0.011903317
GBN32_RS04865	*GBN32_RS04865*	1.115122568	0.011638462
GBN32_RS04815	*GBN32_RS04815*	1.160852661	0.013613269
GBN32_RS04635	*GBN32_RS04635*	1.274162382	0.00839813
GBN32_RS04600	*GBN32_RS04600*	1.131624658	0.016052716
GBN32_RS04595	*GBN32_RS04595*	1.048417035	0.046991593
GBN32_RS04495	*cueR*	1.595384185	0.001237087
GBN32_RS04330	*GBN32_RS04330*	1.839260885	0.000596735
GBN32_RS04275	*lolB*	1.046356071	0.031516168
GBN32_RS04255	*pth*	1.334174519	0.006728098
GBN32_RS04235	*yqfB*	1.130133355	0.010357653
GBN32_RS04225	*GBN32_RS04225*	1.376977271	0.006244209
GBN32_RS04075	*GBN32_RS04075*	1.298635143	0.003830861
GBN32_RS04060	*GBN32_RS04060*	1.632188561	0.001727643
GBN32_RS04005	*GBN32_RS04005*	1.763339726	0.033376343
GBN32_RS04000	*GBN32_RS04000*	1.30877178	0.003220918
GBN32_RS03860	*GBN32_RS03860*	1.01144259	0.040725658
GBN32_RS03775	*GBN32_RS03775*	1.264983016	0.002772185
GBN32_RS03575	*fliJ*	1.618318612	0.000453598
GBN32_RS03570	*fliI*	1.20710617	0.008104613
GBN32_RS03565	*GBN32_RS03565*	1.465775195	0.001664484
GBN32_RS03555	*fliF*	2.044493763	4.76E-06
GBN32_RS03550	*fliE*	3.286913941	3.29E-12
GBN32_RS03540	*GBN32_RS03540*	1.510970576	0.000410901
GBN32_RS03235	*GBN32_RS03235*	1.186717622	0.007049417
GBN32_RS03230	*GBN32_RS03230*	1.083183243	0.013353993
GBN32_RS03145	*GBN32_RS03145*	1.130498585	0.040756224
GBN32_RS03115	*GBN32_RS03115*	1.030443462	0.021880076
GBN32_RS03100	*hybO*	1.064055287	0.015607816
GBN32_RS03090	*hybB*	1.286798304	0.00321639
GBN32_RS02870	*GBN32_RS02870*	1.155105533	0.03592403
GBN32_RS02810	*GBN32_RS02810*	1.13485543	0.010983659

GBN32_RS02770	*ybcJ*	1.079453103	0.01342873
GBN32_RS02765	*GBN32_RS02765*	1.805759361	0.000773721
GBN32_RS02755	*GBN32_RS02755*	1.100399465	0.009598043
GBN32_RS02700	*GBN32_RS02700*	1.049684864	0.017715012
GBN32_RS02695	*GBN32_RS02695*	1.174910698	0.008541834
GBN32_RS02630	*GBN32_RS02630*	1.035583236	0.033490385
GBN32_RS02495	*GBN32_RS02495*	1.001992212	0.017554884
GBN32_RS02480	*dmsD*	1.323404476	0.003163095
GBN32_RS01880	*GBN32_RS01880*	1.56926956	0.001222965
GBN32_RS01800	*GBN32_RS01800*	1.324576662	0.002254211
GBN32_RS01795	*GBN32_RS01795*	1.489948518	0.004269364
GBN32_RS01740	*GBN32_RS01740*	1.543165372	0.00753465
GBN32_RS01625	*GBN32_RS01625*	1.051417762	0.013267985
GBN32_RS01270	*macA*	1.148611221	0.010002315
GBN32_RS01265	*GBN32_RS01265*	1.220287027	0.005357043
GBN32_RS01255	*GBN32_RS01255*	1.473635686	0.000480111
GBN32_RS01250	*GBN32_RS01250*	1.123337785	0.007971917
GBN32_RS01225	*GBN32_RS01225*	1.367076152	0.016452689
GBN32_RS01200	*GBN32_RS01200*	1.205620527	0.007249111
GBN32_RS01165	*GBN32_RS01165*	1.973655005	4.71E-06
GBN32_RS00895	*GBN32_RS00895*	1.079290703	0.01370726
GBN32_RS00810	*GBN32_RS00810*	1.958501033	0.001169549
GBN32_RS00800	*ivy*	1.188890214	0.007151145
GBN32_RS00425	*GBN32_RS00425*	1.061246218	0.01926919
GBN32_RS00180	*GBN32_RS00180*	1.8030727	0.00091912
GBN32_RS00145	*GBN32_RS00145*	1.726176811	0.000853218

Table S7 Transcriptome revealed differential expressed genes related to FliA of the *P. shigelloides*

Gene id	Gene name	log2FoldChange	pvalue
GBN32_RS00050	*GBN32_RS00050*	-4.2192064	4.51E-14
GBN32_RS00160	*GBN32_RS00160*	-1.716714095	0.001934001
GBN32_RS00165	*GBN32_RS00165*	-1.16185484	0.00583914
GBN32_RS00890	*GBN32_RS00890*	-1.475860622	0.001160242
GBN32_RS00910	*sctC*	-1.493567007	0.007183936
GBN32_RS00995	*GBN32_RS00995*	-2.325428819	0.001998931
GBN32_RS01015	*GBN32_RS01015*	-1.420797536	0.000827669
GBN32_RS01030	*GBN32_RS01030*	-1.615748781	0.000181666
GBN32_RS01035	*GBN32_RS01035*	-1.18790882	0.005110128
GBN32_RS01040	*GBN32_RS01040*	-1.366790868	0.001289099

GBN32_RS01055	*GBN32_RS01055*	-1.524819886	0.00032777
GBN32_RS01060	*GBN32_RS01060*	-2.029199668	2.80E-06
GBN32_RS01070	*GBN32_RS01070*	-1.712868355	8.74E-05
GBN32_RS01075	*GBN32_RS01075*	-1.929917693	1.52E-05
GBN32_RS01080	*GBN32_RS01080*	-2.621537057	2.32E-08
GBN32_RS01095	*GBN32_RS01095*	-1.586482922	0.000262466
GBN32_RS01100	*GBN32_RS01100*	-2.277321679	6.53E-07
GBN32_RS01495	*GBN32_RS01495*	-1.855441668	1.84E-05
GBN32_RS01555	*GBN32_RS01555*	-1.415850724	0.004258494
GBN32_RS01615	*folM*	-1.544439204	0.000806979
GBN32_RS01665	*GBN32_RS01665*	-1.554713611	0.00753465
GBN32_RS01680	*GBN32_RS01680*	-1.315242013	0.002621329
GBN32_RS01690	*prpC*	-1.273070244	0.003427447
GBN32_RS01715	*GBN32_RS01715*	-1.333235783	0.003708182
GBN32_RS01765	*GBN32_RS01765*	-1.617573173	0.000148192
GBN32_RS01800	*GBN32_RS01800*	-1.392490475	0.001184807
GBN32_RS01805	*GBN32_RS01805*	-1.598134905	0.000153387
GBN32_RS01850	*GBN32_RS01850*	-1.344569581	0.001851816
GBN32_RS01855	*GBN32_RS01855*	-1.243814098	0.004922105
GBN32_RS01870	*pfkB*	-2.061965469	2.61E-06
GBN32_RS02175	*purD*	-1.247405539	0.005184251
GBN32_RS02215	*rsd*	-2.213537894	3.71E-07
GBN32_RS02385	*putA*	-4.201648774	5.51E-19
GBN32_RS02715	*GBN32_RS02715*	-1.989360554	1.45E-05
GBN32_RS02740	*GBN32_RS02740*	-3.037245433	0.003515158
GBN32_RS02835	*nrfD*	-1.333671455	0.002210575
GBN32_RS02850	*GBN32_RS02850*	-1.279972363	0.005343688
GBN32_RS02855	*GBN32_RS02855*	-2.080295577	2.50E-06
GBN32_RS02930	*GBN32_RS02930*	-1.573426449	0.000213284
GBN32_RS02935	*htpX*	-1.593111786	0.000212689
GBN32_RS02965	*GBN32_RS02965*	-1.401745785	0.00087091
GBN32_RS02990	*GBN32_RS02990*	-1.359925589	0.00127708
GBN32_RS02995	*GBN32_RS02995*	-1.330891561	0.001552855
GBN32_RS03000	*GBN32_RS03000*	-1.667726122	0.00012691
GBN32_RS03005	*GBN32_RS03005*	-2.618499746	2.61E-09
GBN32_RS03085	*hybC*	-2.550783865	9.44E-09
GBN32_RS03090	*hybB*	-2.646786747	2.81E-08
GBN32_RS03095	*hybA*	-1.791274531	0.00010947
GBN32_RS03175	*GBN32_RS03175*	-1.300672688	0.002091048
GBN32_RS03195	*GBN32_RS03195*	-1.953682328	9.21E-06
GBN32_RS03240	*pagP*	-2.397918114	1.15E-07
GBN32_RS03375	*flgN*	-1.344379784	0.002493106
GBN32_RS03380	*flgM*	-2.751463393	8.69E-10
GBN32_RS03290	*cheV*	-1.402331777	0.007766615

GBN32_RS03395	*cheR*	-1.339290575	0.002672102
GBN32_RS03460	*flaC*	-2.92055002	1.22E-10
GBN32_RS03515	*flaG*	-5.547532583	7.04E-28
GBN32_RS03520	*flaH*	-2.024669343	2.93E-05
GBN32_RS03525	*fliD*	-1.369710211	0.00724203
GBN32_RS03645	*GBN32_RS03645*	-12.27153963	7.71E-32
GBN32_RS03725	*GBN32_RS03725*	-1.237211801	0.00384546
GBN32_RS03750	*tnaA*	-1.758518914	3.13E-05
GBN32_RS03755	*GBN32_RS03755*	-2.024911357	3.18E-06
GBN32_RS03765	*GBN32_RS03765*	-2.116036205	3.76E-05
GBN32_RS03770	*GBN32_RS03770*	-1.361831657	0.0011249
GBN32_RS03785	*fadI*	-2.077854665	1.23E-06
GBN32_RS03790	*fadJ*	-1.302746624	0.001826116
GBN32_RS03925	*GBN32_RS03925*	-1.335042204	0.00153298
GBN32_RS03980	*purF*	-1.16514989	0.005853883
GBN32_RS03985	*GBN32_RS03985*	-1.503952475	0.001154997
GBN32_RS04120	*GBN32_RS04120*	-2.246373566	8.12E-07
GBN32_RS04130	*GBN32_RS04130*	-1.228936026	0.003470119
GBN32_RS04165	*nqrM*	-1.333098453	0.007859683
GBN32_RS04170	*GBN32_RS04170*	-1.421516451	0.002690277
GBN32_RS04175	*GBN32_RS04175*	-1.323878126	0.00498103
GBN32_RS04310	*GBN32_RS04310*	-1.407510847	0.000885841
GBN32_RS04480	*GBN32_RS04480*	-1.212301441	0.005033618
GBN32_RS04525	*folX*	-1.151157436	0.006297971
GBN32_RS04615	*GBN32_RS04615*	-2.837286688	1.06E-07
GBN32_RS04640	*GBN32_RS04640*	-1.490188025	0.001087016
GBN32_RS04680	*hspQ*	-1.906226286	2.07E-05
GBN32_RS04700	*GBN32_RS04700*	-1.164630564	0.005265012
GBN32_RS04720	*napF*	-1.856649754	0.000177657
GBN32_RS04725	*napD*	-2.914487108	0.001173602
GBN32_RS04730	*napA*	-2.070962441	3.66E-06
GBN32_RS04735	*napG*	-2.946947312	2.01E-09
GBN32_RS04740	*napH*	-1.733023844	0.000483794
GBN32_RS04745	*napB*	-2.194129482	7.47E-06
GBN32_RS04750	*napC*	-1.727877208	0.000124996
GBN32_RS04760	*GBN32_RS04760*	-1.365843234	0.002876003
GBN32_RS04765	*GBN32_RS04765*	-2.183484241	3.68E-06
GBN32_RS04770	*GBN32_RS04770*	-1.717428157	5.01E-05
GBN32_RS04775	*GBN32_RS04775*	-1.838745021	2.81E-05
GBN32_RS04780	*GBN32_RS04780*	-1.870456647	2.52E-05
GBN32_RS04785	*GBN32_RS04785*	-1.691926386	9.98E-05
GBN32_RS04790	*GBN32_RS04790*	-1.455348065	0.000723861
GBN32_RS04995	*panM*	-1.166571767	0.006457511
GBN32_RS05015	*livG*	-1.605378582	0.006984897

GBN32_RS05045	*glpD*	-1.606290914	0.000134904
GBN32_RS05100	*GBN32_RS05100*	-2.223510092	2.39E-05
GBN32_RS05170	*gspH*	-1.281205373	0.004610497
GBN32_RS05295	*GBN32_RS05295*	-1.47388835	0.001787405
GBN32_RS05330	*GBN32_RS05330*	-3.405682299	7.12E-14
GBN32_RS05335	*astA*	-2.317566114	1.82E-07
GBN32_RS05465	*GBN32_RS05465*	-1.3464881	0.001564021
GBN32_RS05515	*GBN32_RS05515*	-2.29904976	1.79E-07
GBN32_RS05520	*GBN32_RS05520*	-2.041018045	2.89E-06
GBN32_RS05615	*putP*	-3.068918522	2.23E-11
GBN32_RS05730	*fkpB*	-1.258298237	0.003384332
GBN32_RS06735	*petA*	-2.288149161	1.41E-07
GBN32_RS06740	*GBN32_RS06740*	-1.835406765	1.80E-05
GBN32_RS06745	*GBN32_RS06745*	-2.384901094	4.33E-08
GBN32_RS06885	*mutT*	-1.688809814	0.000256428
GBN32_RS06970	*GBN32_RS06970*	-2.778403652	8.79E-08
GBN32_RS07010	*acnB*	-1.879007808	9.80E-06
GBN32_RS07200	*GBN32_RS07200*	-1.446074323	0.001761843
GBN32_RS07230	*GBN32_RS07230*	-1.66074185	0.005032069
GBN32_RS07285	*pyrI*	-2.851184371	1.35E-09
GBN32_RS07290	*pyrB*	-2.360830623	1.88E-07
GBN32_RS07395	*dnaK*	-2.410006874	2.43E-08
GBN32_RS07675	*ssrS*	-2.326171585	7.29E-08
GBN32_RS07705	*gcvT*	-1.878224054	1.10E-05
GBN32_RS07710	*gcvH*	-1.829365002	1.70E-05
GBN32_RS07730	*GBN32_RS07730*	-1.314879592	0.003059028
GBN32_RS07800	*era*	-1.120621052	0.007702272
GBN32_RS07885	*nlpD*	-1.307373041	0.001741505
GBN32_RS07890	*rpoS*	-1.299885603	0.001840961
GBN32_RS07935	*GBN32_RS07935*	-11.42377613	6.31E-26
GBN32_RS07940	*GBN32_RS07940*	-9.166120562	2.40E-12
GBN32_RS08130	*thiL*	-1.695900991	0.000104953
GBN32_RS08155	*pomA*	-3.517554555	4.25E-11
GBN32_RS08160	*GBN32_RS08160*	-2.749513591	1.68E-08
GBN32_RS08195	*GBN32_RS08195*	-2.164857822	8.96E-06
GBN32_RS08300	*GBN32_RS08300*	-2.116688146	0.000156638
GBN32_RS08305	*GBN32_RS08305*	-1.466501859	0.00153115
GBN32_RS08310	*GBN32_RS08310*	-1.893453853	9.80E-05
GBN32_RS08340	*mrdB*	-1.331576769	0.003511264
GBN32_RS08425	*GBN32_RS08425*	-1.451995282	0.007068652
GBN32_RS08505	*GBN32_RS08505*	-1.716675557	0.000208475
GBN32_RS08565	*GBN32_RS08565*	-1.273486798	0.00225895
GBN32_RS08570	*sdhC*	-2.541302861	6.24E-09
GBN32_RS08575	*sdhD*	-3.238117715	8.82E-12

GBN32_RS08580	*sdhA*	-2.587621687	3.37E-09
GBN32_RS08585	*GBN32_RS08585*	-3.045039771	7.00E-12
GBN32_RS08590	*sucA*	-1.384392425	0.000923623
GBN32_RS08595	*odhB*	-1.294299807	0.00191472
GBN32_RS08620	*znuA*	-1.125982708	0.007072997
GBN32_RS08655	*GBN32_RS08655*	-1.270510025	0.003326873
GBN32_RS08715	*GBN32_RS08715*	-4.626241575	9.22E-22
GBN32_RS08720	*GBN32_RS08720*	-2.593050114	2.73E-09
GBN32_RS08875	*GBN32_RS08875*	-1.939421227	0.000157538
GBN32_RS08880	*fadD*	-2.727234952	5.55E-10
GBN32_RS08950	*GBN32_RS08950*	-4.025214175	9.63E-18
GBN32_RS08955	*GBN32_RS08955*	-4.291502809	3.78E-19
GBN32_RS08960	*GBN32_RS08960*	-4.75041473	1.06E-19
GBN32_RS08965	*GBN32_RS08965*	-4.352919315	4.21E-20
GBN32_RS09000	*GBN32_RS09000*	-2.576886595	4.49E-09
GBN32_RS09005	*GBN32_RS09005*	-1.726897361	5.72E-05
GBN32_RS09030	*GBN32_RS09030*	-1.819827106	0.000306115
GBN32_RS09035	*GBN32_RS09035*	-1.950369441	9.98E-06
GBN32_RS09065	*GBN32_RS09065*	-2.65873123	7.74E-06
GBN32_RS09075	*GBN32_RS09075*	-2.480374415	1.25E-07
GBN32_RS09095	*GBN32_RS09095*	-2.539525272	7.15E-09
GBN32_RS09245	*rsmS*	-1.743765529	0.000152113
GBN32_RS09250	*GBN32_RS09250*	-2.459764833	5.55E-08
GBN32_RS09305	*htpG*	-2.116860738	7.66E-07
GBN32_RS09350	*lon*	-1.351492536	0.001244099
GBN32_RS09390	*GBN32_RS09390*	-2.820591825	1.25E-09
GBN32_RS09400	*GBN32_RS09400*	-1.228473657	0.003850556
GBN32_RS09570	*GBN32_RS09570*	-2.175335183	8.08E-07
GBN32_RS09585	*hisC*	-1.226914132	0.003589383
GBN32_RS09660	*msrP*	-1.514447966	0.000649536
GBN32_RS09735	*GBN32_RS09735*	-1.720124354	0.004015113
GBN32_RS09745	*GBN32_RS09745*	-2.95967442	7.02E-05
GBN32_RS09750	*GBN32_RS09750*	-2.187399276	0.000201115
GBN32_RS09755	*GBN32_RS09755*	-2.863296228	2.81E-07
GBN32_RS09790	*GBN32_RS09790*	-1.813872852	2.64E-05
GBN32_RS09865	*GBN32_RS09865*	-2.950434361	0.005247305
GBN32_RS09925	*GBN32_RS09925*	-1.329243154	0.001542029
GBN32_RS09930	*gntR*	-1.14980439	0.006399439
GBN32_RS09995	*cadA*	-3.085777234	1.65E-11
GBN32_RS10000	*cadB*	-2.585354085	3.07E-08
GBN32_RS10410	*GBN32_RS10410*	-1.76149952	4.18E-05
GBN32_RS10465	*tssG*	-1.484369563	0.000469352
GBN32_RS10685	*gbpA*	-1.502832843	0.002565531
GBN32_RS10710	*glsA*	-3.640947975	8.78E-15

GBN32_RS10715	*GBN32_RS10715*	-3.159971516	6.05E-12
GBN32_RS10720	*GBN32_RS10720*	-2.799236411	5.39E-07
GBN32_RS10725	*GBN32_RS10725*	-3.399910215	9.34E-13
GBN32_RS10735	*GBN32_RS10735*	-1.91187699	1.34E-05
GBN32_RS10740	*GBN32_RS10740*	-1.641432808	0.00017834
GBN32_RS10745	*GBN32_RS10745*	-1.297673534	0.002134787
GBN32_RS10770	*GBN32_RS10770*	-1.267907877	0.005976746
GBN32_RS10835	*GBN32_RS10835*	-1.248256698	0.002786456
GBN32_RS10845	*nhaC*	-3.660824403	1.44E-13
GBN32_RS10850	*arcA*	-2.116361189	1.87E-06
GBN32_RS10865	*GBN32_RS10865*	-3.274708519	6.87E-09
GBN32_RS10875	*ydfZ*	-1.590318922	0.000203514
GBN32_RS10880	*GBN32_RS10880*	-3.944884913	1.47E-05
GBN32_RS11220	*sthA*	-2.062686827	1.44E-06
GBN32_RS11275	*GBN32_RS11275*	-1.608731669	0.001837153
GBN32_RS11330	*hslV*	-1.506586989	0.000419345
GBN32_RS11335	*hslU*	-1.1423148	0.006288183
GBN32_RS11355	*GBN32_RS11355*	-1.586638842	0.000166943
GBN32_RS11360	*glpK*	-1.810795369	1.87E-05
GBN32_RS11470	*GBN32_RS11470*	-1.916629623	1.66E-05
GBN32_RS11610	*pyrE*	-2.061997335	7.87E-05
GBN32_RS11635	*GBN32_RS11635*	-2.212136568	0.00033982
GBN32_RS11650	*GBN32_RS11650*	-4.536417361	1.78E-19
GBN32_RS11770	*GBN32_RS11770*	-1.796273593	2.38E-05
GBN32_RS11835	*prlC*	-1.560842997	0.000216367
GBN32_RS12075	*GBN32_RS12075*	-1.877105355	1.34E-05
GBN32_RS12095	*glpQ*	-1.697743071	5.86E-05
GBN32_RS12135	*GBN32_RS12135*	-2.222087581	4.78E-05
GBN32_RS12175	*GBN32_RS12175*	-1.198031472	0.007770354
GBN32_RS12195	*GBN32_RS12195*	-2.445468116	2.39E-08
GBN32_RS12200	*GBN32_RS12200*	-1.513414174	0.0003225
GBN32_RS12390	*GBN32_RS12390*	-2.108034379	2.59E-06
GBN32_RS12450	*GBN32_RS12450*	-1.582797905	0.000280005
GBN32_RS12555	*bcsB*	-1.101414766	0.008355622
GBN32_RS12760	*GBN32_RS12760*	-1.741770709	0.000188622
GBN32_RS12815	*glmS*	-1.453175887	0.000545088
GBN32_RS12820	*GBN32_RS12820*	-1.551528738	0.000240033
GBN32_RS12990	*GBN32_RS12990*	-1.824783517	1.80E-05
GBN32_RS13005	*fadA*	-1.99781657	2.86E-06
GBN32_RS13010	*fadB*	-2.772598988	2.61E-10
GBN32_RS13050	*GBN32_RS13050*	-1.534859464	0.00181487
GBN32_RS13055	*GBN32_RS13055*	-1.518383417	0.000711439
GBN32_RS13060	*rffM*	-1.907208697	1.12E-05
GBN32_RS13185	*GBN32_RS13185*	-1.666474413	0.00010268

GBN32_RS13275	*GBN32_RS13275*	-1.430720077	0.004450165
GBN32_RS13280	*GBN32_RS13280*	-1.36799009	0.003963599
GBN32_RS13360	*GBN32_RS13360*	-2.15500473	1.16E-06
GBN32_RS13365	*GBN32_RS13365*	-4.380037777	4.45E-17
GBN32_RS13420	*csgD*	-3.53755672	1.07E-12
GBN32_RS13425	*csgE*	-2.800664151	1.14E-08
GBN32_RS13430	*GBN32_RS13430*	-1.762056254	6.13E-05
GBN32_RS13435	*GBN32_RS13435*	-1.295154376	0.002511291
GBN32_RS13450	*GBN32_RS13450*	-2.898526579	1.99E-08
GBN32_RS13455	*GBN32_RS13455*	-1.361398838	0.001182977
GBN32_RS13530	*GBN32_RS13530*	-2.151979414	3.34E-06
GBN32_RS13540	*GBN32_RS13540*	-3.635800416	9.35E-06
GBN32_RS13580	*rsxG*	-1.481667631	0.000918039
GBN32_RS13585	*GBN32_RS13585*	-1.182924703	0.005779739
GBN32_RS13600	*GBN32_RS13600*	-2.135667257	2.30E-05
GBN32_RS13680	*cspD*	-1.369369389	0.001063996
GBN32_RS13755	*GBN32_RS13755*	-2.212244263	3.72E-07
GBN32_RS13760	*GBN32_RS13760*	-3.359050612	8.82E-14
GBN32_RS13820	*GBN32_RS13820*	-5.532623525	3.56E-27
GBN32_RS14025	*GBN32_RS14025*	-1.328650676	0.007168988
GBN32_RS14110	*GBN32_RS14110*	-1.135064439	0.006870285
GBN32_RS14165	*GBN32_RS14165*	-2.548222636	8.56E-09
GBN32_RS14170	*GBN32_RS14170*	-1.523303825	0.000296419
GBN32_RS14175	*groL*	-1.200885248	0.003905602
GBN32_RS14180	*GBN32_RS14180*	-1.168268567	0.005876503
GBN32_RS14220	*frdD*	-1.904103184	7.47E-06
GBN32_RS14225	*frdC*	-1.712087019	5.13E-05
GBN32_RS14230	*GBN32_RS14230*	-1.210805414	0.003655621
GBN32_RS14350	*GBN32_RS14350*	-5.007535629	4.69E-18
GBN32_RS14505	*GBN32_RS14505*	-1.342490794	0.003037937
GBN32_RS14510	*GBN32_RS14510*	-1.281067234	0.002476318
GBN32_RS14685	*GBN32_RS14685*	-1.28248795	0.002121576
GBN32_RS14715	*GBN32_RS14715*	-2.011559005	8.97E-06
GBN32_RS14895	*GBN32_RS14895*	-2.453767814	3.80E-08
GBN32_RS14910	*GBN32_RS14910*	-2.203433329	8.72E-07
GBN32_RS14960	*recJ*	-1.3683327	0.001079823
GBN32_RS15155	*GBN32_RS15155*	-1.487399578	0.000499519
GBN32_RS15300	*GBN32_RS15300*	-2.223398048	2.26E-07
GBN32_RS15440	*GBN32_RS15440*	-1.454073915	0.001467964
GBN32_RS15450	*GBN32_RS15450*	-2.314996156	2.90E-06
GBN32_RS15465	*yejB*	-1.269408277	0.004195177
GBN32_RS15515	*GBN32_RS15515*	-1.399889993	0.001207773
GBN32_RS15520	*GBN32_RS15520*	-1.846428999	8.07E-05
GBN32_RS15530	*GBN32_RS15530*	-1.733661216	0.000231443

GBN32_RS15535	*GBN32_RS15535*	-4.386081211	4.91E-18
GBN32_RS15560	*GBN32_RS15560*	-1.370822461	0.001291885
GBN32_RS15700	*GBN32_RS15700*	-1.355176333	0.002308659
GBN32_RS15855	*GBN32_RS15855*	-3.187445743	4.17E-11
GBN32_RS15860	*fadE*	-3.293713768	5.65E-13
GBN32_RS15885	*aceB*	-3.007252638	1.39E-11
GBN32_RS15890	*aceA*	-1.524098722	0.000281627
GBN32_RS16065	*GBN32_RS16065*	-1.567099792	0.000225084
GBN32_RS16205	*icd*	-1.386233962	0.000917933
GBN32_RS16255	*GBN32_RS16255*	-1.61813069	0.000193954
GBN32_RS16275	*GBN32_RS16275*	-2.696406096	2.97E-09
GBN32_RS16310	*ppsA*	-1.861267438	1.44E-05
GBN32_RS16315	*GBN32_RS16315*	-1.250395829	0.003831986
GBN32_RS16365	*GBN32_RS16365*	-1.426579297	0.000702572
GBN32_RS16370	*GBN32_RS16370*	-1.641897676	0.000114351
GBN32_RS16375	*GBN32_RS16375*	-2.131420729	3.58E-06
GBN32_RS16440	*GBN32_RS16440*	-2.541445033	9.06E-05
GBN32_RS16525	*GBN32_RS16525*	-1.967703087	1.89E-05
GBN32_RS16605	*ccoN*	-1.493771707	0.000412468
GBN32_RS16610	*ccoO*	-1.543481054	0.000305905
GBN32_RS16615	*GBN32_RS16615*	-1.689390449	0.000174102
GBN32_RS16620	*ccoP*	-1.929220691	7.83E-06
GBN32_RS16625	*GBN32_RS16625*	-2.34552553	2.34E-07
GBN32_RS16635	*ccoS*	-1.314677354	0.008191344
GBN32_RS16640	*GBN32_RS16640*	-1.616738175	0.000208622
GBN32_RS16780	*GBN32_RS16780*	-1.967592414	1.73E-05
GBN32_RS16980	*GBN32_RS16980*	-1.426966419	0.004294507
GBN32_RS17055	*astB*	-1.446780127	0.000691605
GBN32_RS17105	*GBN32_RS17105*	-2.369334004	0.000860935
GBN32_RS17165	*GBN32_RS17165*	-1.42840922	0.002483067
GBN32_RS17215	*nuoF*	-1.579748682	0.000181595
GBN32_RS17220	*nuoE*	-1.551161629	0.000272636
GBN32_RS17225	*nuoC*	-1.241294263	0.00308014
GBN32_RS17230	*GBN32_RS17230*	-1.395959828	0.000891277
GBN32_RS17235	*GBN32_RS17235*	-1.530733518	0.000325456
GBN32_RS17280	*GBN32_RS17280*	-1.989134466	0.001239507
GBN32_RS17450	*GBN32_RS17450*	-2.508733476	4.99E-07
GBN32_RS17455	*GBN32_RS17455*	-2.102183212	9.66E-07
GBN32_RS17475	*GBN32_RS17475*	-1.276775019	0.002415623
GBN32_RS17550	*GBN32_RS17550*	-1.882831604	3.01E-05
GBN32_RS17555	*GBN32_RS17555*	-2.8093905	3.00E-10
GBN32_RS17560	*GBN32_RS17560*	-2.016903328	2.88E-06
GBN32_RS17730	*GBN32_RS17730*	-1.201463661	0.006810396
GBN32_RS17750	*GBN32_RS17750*	-1.292699105	0.002178205

GBN32_RS17900	*GBN32_RS17900*	-1.341449124	0.001396395
GBN32_RS17905	*hypF*	-1.383732299	0.001016462
GBN32_RS17935	*GBN32_RS17935*	-2.148372588	1.78E-05
GBN32_RS00745	*GBN32_RS00745*	1.651148895	0.000296014
GBN32_RS00750	*GBN32_RS00750*	5.103489266	7.88E-24
GBN32_RS00755	*GBN32_RS00755*	2.758792016	6.24E-08
GBN32_RS00765	*GBN32_RS00765*	2.665957746	3.14E-09
GBN32_RS00810	*GBN32_RS00810*	2.224626546	1.11E-06
GBN32_RS00985	*sctA*	2.130896399	0.000411285
GBN32_RS01165	*GBN32_RS01165*	1.85749967	0.000161757
GBN32_RS01170	*GBN32_RS01170*	1.175111035	0.00755622
GBN32_RS01220	*GBN32_RS01220*	1.78949043	2.51E-05
GBN32_RS01450	*thrS*	1.300110452	0.001830489
GBN32_RS01455	*infC*	1.115722117	0.007234351
GBN32_RS01465	*rplT*	1.195078549	0.004125715
GBN32_RS02280	*GBN32_RS02280*	1.374621737	0.003599348
GBN32_RS02300	*metA*	1.399833683	0.00183756
GBN32_RS02785	*cfa*	1.293819938	0.001936495
GBN32_RS02865	*purR*	1.133501161	0.006692578
GBN32_RS02910	*GBN32_RS02910*	1.52542876	0.000288892
GBN32_RS03120	*GBN32_RS03120*	1.646207016	9.91E-05
GBN32_RS03130	*GBN32_RS03130*	1.167008995	0.004997182
GBN32_RS03180	*treC*	5.908491456	4.82E-31
GBN32_RS03185	*treB*	6.128208682	2.49E-32
GBN32_RS03230	*GBN32_RS03230*	2.008404679	3.07E-06
GBN32_RS03310	*dbpA*	1.789840875	3.89E-05
GBN32_RS03360	*GBN32_RS03360*	1.791831618	2.60E-05
GBN32_RS03400	*flgB*	3.427144785	6.08E-14
GBN32_RS03405	*flgC*	3.415505829	6.79E-14
GBN32_RS03410	*GBN32_RS03410*	3.258578312	6.48E-13
GBN32_RS03415	*flgE*	1.920205331	6.69E-06
GBN32_RS03420	*GBN32_RS03420*	1.138194314	0.006930418
GBN32_RS03425	*flgF*	1.276296916	0.002441509
GBN32_RS03430	*flgG*	1.414013129	0.000776608
GBN32_RS03435	*GBN32_RS03435*	1.564680792	0.000241533
GBN32_RS03440	*flgJ*	1.279804248	0.002547159
GBN32_RS03445	*flgH*	1.588153291	0.000208776
GBN32_RS03685	*GBN32_RS03685*	3.116598348	5.49E-12
GBN32_RS03695	*iolD*	1.796354967	3.71E-05
GBN32_RS03710	*GBN32_RS03710*	2.585457939	6.07E-08
GBN32_RS03730	*iolC*	2.801519457	3.98E-10
GBN32_RS03735	*iolE*	3.149649038	1.55E-12
GBN32_RS03740	*iolB*	3.780735618	7.45E-16
GBN32_RS03775	*GBN32_RS03775*	1.321852744	0.00157357

GBN32_RS04010	*GBN32_RS04010*	1.296060676	0.002043099
GBN32_RS04135	*GBN32_RS04135*	2.623476905	1.43E-08
GBN32_RS04140	*GBN32_RS04140*	1.461666342	0.000708065
GBN32_RS04225	*GBN32_RS04225*	1.567758102	0.000417803
GBN32_RS04280	*hemA*	1.117322791	0.007688365
GBN32_RS04285	*prfA*	1.762520341	4.06E-05
GBN32_RS04320	*GBN32_RS04320*	1.892130388	1.95E-05
GBN32_RS04335	*GBN32_RS04335*	1.431668315	0.000645599
GBN32_RS04340	*ybaK*	1.353563326	0.001207494
GBN32_RS04390	*GBN32_RS04390*	1.493380615	0.000809256
GBN32_RS04415	*GBN32_RS04415*	1.803906972	3.12E-05
GBN32_RS04550	*oppB*	1.264361229	0.002674968
GBN32_RS04555	*oppC*	1.130525208	0.007425276
GBN32_RS04960	*GBN32_RS04960*	1.145693091	0.006646075
GBN32_RS04965	*GBN32_RS04965*	1.214738942	0.003880452
GBN32_RS04975	*ftsY*	1.417824768	0.000835355
GBN32_RS04980	*ftsE*	1.185441596	0.005448767
GBN32_RS05105	*envZ*	1.750137523	3.56E-05
GBN32_RS05125	*pckA*	1.996145015	2.73E-06
GBN32_RS05215	*GBN32_RS05215*	1.484240895	0.000477231
GBN32_RS05250	*aroK*	1.216233821	0.003630281
GBN32_RS05255	*aroB*	1.594646171	0.000173456
GBN32_RS05260	*GBN32_RS05260*	1.339804694	0.001519312
GBN32_RS05435	*rpsL*	1.152434137	0.005638087
GBN32_RS05440	*rpsG*	1.326784861	0.001508252
GBN32_RS05550	*GBN32_RS05550*	3.682201568	6.89E-16
GBN32_RS05560	*GBN32_RS05560*	2.234227366	1.79E-06
GBN32_RS05575	*GBN32_RS05575*	1.398863142	0.001117255
GBN32_RS05630	*hcp1*	1.3386637	0.001448265
GBN32_RS05765	*greA*	1.670068477	9.43E-05
GBN32_RS05780	*ftsH*	1.110386054	0.007502741
GBN32_RS05850	*GBN32_RS05850*	1.599056664	0.000166661
GBN32_RS05870	*GBN32_RS05870*	1.352175475	0.001963915
GBN32_RS05875	*GBN32_RS05875*	1.206912913	0.005471331
GBN32_RS05900	*speF*	1.851545045	1.23E-05
GBN32_RS05930	*GBN32_RS05930*	3.923013982	7.57E-17
GBN32_RS05935	*hdcC*	2.257133155	1.81E-07
GBN32_RS06030	*GBN32_RS06030*	4.389934904	4.08E-10
GBN32_RS06085	*ruvX*	1.91823963	3.20E-05
GBN32_RS06090	*GBN32_RS06090*	1.50834121	0.000663645
GBN32_RS06135	*metK*	3.175116566	1.43E-12
GBN32_RS06260	*cadB*	1.8661657	1.16E-05
GBN32_RS06265	*GBN32_RS06265*	2.034408422	1.88E-06
GBN32_RS06300	*proS*	1.513297121	0.000331097

GBN32_RS06390	*GBN32_RS06390*	1.633507607	0.000208027
GBN32_RS06400	*GBN32_RS06400*	1.234989863	0.004229273
GBN32_RS06495	*fis*	1.790388083	6.93E-05
GBN32_RS06500	*dusB*	1.507829234	0.00041042
GBN32_RS07040	*ompA*	2.851882819	8.92E-11
GBN32_RS07210	*GBN32_RS07210*	1.37253769	0.001312527
GBN32_RS07925	*GBN32_RS07925*	1.197975285	0.00427287
GBN32_RS07980	*GBN32_RS07980*	1.164382064	0.005726956
GBN32_RS08085	*GBN32_RS08085*	1.830900005	0.000163612
GBN32_RS08090	*GBN32_RS08090*	2.083136905	1.31E-06
GBN32_RS08105	*proP*	2.055863039	1.72E-06
GBN32_RS08405	*miaB*	1.465644578	0.000554821
GBN32_RS08465	*GBN32_RS08465*	1.158474297	0.006283516
GBN32_RS08540	*GBN32_RS08540*	2.090094266	1.66E-06
GBN32_RS08730	*GBN32_RS08730*	1.887529632	2.30E-05
GBN32_RS09235	*GBN32_RS09235*	2.074457873	1.21E-06
GBN32_RS09420	*ackA*	1.23525547	0.003204061
GBN32_RS09430	*GBN32_RS09430*	1.446236294	0.000665811
GBN32_RS09580	*gndA*	1.242525493	0.002983982
GBN32_RS09740	*gltX*	1.38734317	0.000976116
GBN32_RS09785	*pyrD*	1.259376907	0.004071671
GBN32_RS09935	*GBN32_RS09935*	1.439977276	0.000764174
GBN32_RS10010	*rlmKL*	1.326892741	0.001579393
GBN32_RS10070	*hdcA*	4.40050171	1.79E-20
GBN32_RS10360	*cadA*	1.368970046	0.001045968
GBN32_RS10425	*GBN32_RS10425*	3.423484716	2.89E-13
GBN32_RS10445	*tssB*	1.80232413	2.06E-05
GBN32_RS10450	*tssC*	1.386908728	0.000909663
GBN32_RS10525	*GBN32_RS10525*	1.12773186	0.006784602
GBN32_RS10535	*GBN32_RS10535*	2.897094916	0.005247305
GBN32_RS10555	*GBN32_RS10555*	1.253428626	0.003114961
GBN32_RS10890	*GBN32_RS10890*	1.194288791	0.005982408
GBN32_RS10895	*GBN32_RS10895*	1.487988634	0.000975661
GBN32_RS10990	*rpsJ*	1.573627252	0.000188524
GBN32_RS10995	*rplC*	1.520019061	0.000300793
GBN32_RS11250	*GBN32_RS11250*	1.564719568	0.000247131
GBN32_RS11255	*argB*	1.91828017	1.95E-05
GBN32_RS11260	*argC*	2.24502553	2.66E-07
GBN32_RS11290	*metB*	1.517872466	0.000547897
GBN32_RS11295	*metJ*	1.179434692	0.005679768
GBN32_RS11370	*emrD*	1.382979096	0.003391762
GBN32_RS11395	*tpiA*	1.887290599	8.73E-06
GBN32_RS11620	*GBN32_RS11620*	1.127137453	0.008157163
GBN32_RS11660	*gmk*	1.457574287	0.000563824

GBN32_RS11665	*rpoZ*	1.244516782	0.00310059
GBN32_RS11670	*spoT*	1.501884617	0.000362855
GBN32_RS11775	*yihA*	1.186426289	0.005076023
GBN32_RS11860	*GBN32_RS11860*	1.62157284	0.000156605
GBN32_RS11890	*yigB*	1.396014002	0.002097209
GBN32_RS11895	*xerC*	1.723015233	0.000321737
GBN32_RS11945	*hemX*	1.774013575	2.81E-05
GBN32_RS11950	*GBN32_RS11950*	1.461655305	0.000506958
GBN32_RS12090	*crcB*	1.417737175	0.001445074
GBN32_RS12160	*GBN32_RS12160*	1.483769039	0.000520673
GBN32_RS12205	*GBN32_RS12205*	3.156836738	2.04E-12
GBN32_RS12210	*cydB*	3.322450941	2.92E-13
GBN32_RS12215	*cydX*	1.906080678	7.04E-05
GBN32_RS12270	*potE*	2.305204496	8.37E-08
GBN32_RS12370	*GBN32_RS12370*	1.395474232	0.000893137
GBN32_RS12435	*ilvG*	1.445201998	0.001395573
GBN32_RS12665	*fliD*	1.295222148	0.002220842
GBN32_RS12670	*GBN32_RS12670*	1.50464004	0.000345071
GBN32_RS12680	*flgL*	1.928647757	2.10E-05
GBN32_RS12685	*flgK*	1.779940796	3.29E-05
GBN32_RS12740	*flgM*	1.484667618	0.000532208
GBN32_RS12865	*yidC*	1.433810046	0.000643035
GBN32_RS12945	*adiA*	2.772809065	2.49E-10
GBN32_RS12960	*glyS*	1.140730555	0.006401201
GBN32_RS12965	*glyQ*	1.676852331	8.44E-05
GBN32_RS13025	*trkH*	1.46907293	0.000892257
GBN32_RS13120	*rho*	1.112440234	0.007432907
GBN32_RS13165	*ubiB*	1.171530316	0.00527618
GBN32_RS13350	*nadA*	2.03097008	1.75E-05
GBN32_RS13355	*GBN32_RS13355*	2.113024202	1.23E-06
GBN32_RS13475	*moeA*	1.156069359	0.006019911
GBN32_RS13480	*moeB*	1.610102837	0.00027997
GBN32_RS13535	*GBN32_RS13535*	1.640885169	0.000323155
GBN32_RS13660	*GBN32_RS13660*	1.291052922	0.002164389
GBN32_RS13665	*infA*	1.579236421	0.000296323
GBN32_RS13685	*GBN32_RS13685*	1.241135692	0.003211933
GBN32_RS13810	*GBN32_RS13810*	1.136721356	0.006243623
GBN32_RS13880	*GBN32_RS13880*	6.212355004	2.09E-31
GBN32_RS14705	*GBN32_RS14705*	1.321902213	0.001555179
GBN32_RS14800	*GBN32_RS14800*	1.209299849	0.007895856
GBN32_RS14930	*srmB*	1.489755072	0.000427576
GBN32_RS14935	*GBN32_RS14935*	1.496362961	0.004879682
GBN32_RS14940	*brnQ*	1.151587263	0.007272118
GBN32_RS15120	*GBN32_RS15120*	1.521536783	0.000469634

GBN32_RS15140	*GBN32_RS15140*	1.497666366	0.000781555
GBN32_RS15145	*nrfB*	1.350061815	0.001364653
GBN32_RS15165	*GBN32_RS15165*	2.001673069	4.89E-06
GBN32_RS15480	*GBN32_RS15480*	1.389601661	0.000883331
GBN32_RS15730	*deoA*	1.541616765	0.000248982
GBN32_RS15940	*iscR*	1.464921155	0.00055191
GBN32_RS16040	*xseA*	1.376981607	0.001066287
GBN32_RS16070	*mltF*	1.16604814	0.0052325
GBN32_RS16290	*zntB*	1.34157126	0.002018378
GBN32_RS16350	*GBN32_RS16350*	4.838940612	9.72E-21
GBN32_RS16355	*GBN32_RS16355*	3.746491805	6.90E-16
GBN32_RS16410	*cobB*	1.31794098	0.001763113
GBN32_RS16495	*pfkB*	1.379152477	0.001740743
GBN32_RS16745	*hchA*	1.157743664	0.005409334
GBN32_RS16790	*ptsG*	1.181889802	0.004503789
GBN32_RS16820	*fabF*	1.330846338	0.001537031
GBN32_RS16990	*pykF*	1.919026396	6.61E-06
GBN32_RS17680	*GBN32_RS17680*	1.473804334	0.000581127
GBN32_RS17685	*GBN32_RS17685*	1.493047464	0.000404671
GBN32_RS17975	*GBN32_RS17975*	2.635529091	5.55E-06
GBN32_RS18110	*GBN32_RS18110*	1.399312904	0.001503435

Table S8 Transcriptome revealed differential expressed genes related to FliA$_L$ of the *P. shigelloides*

Gene id	Gene name	log2FoldChange	pvalue
GBN32_RS04745	*napB*	-1.623600063	0.000529718
GBN32_RS04750	*napC*	-1.565647037	0.000452742
GBN32_RS10465	*tssG*	-1.611301553	0.000156457
GBN32_RS10470	*tagH*	-1.728091898	4.91E-05
GBN32_RS10475	*tssJ*	-1.842325381	3.56E-05
GBN32_RS10480	*tssK*	-1.718973729	5.45E-05
GBN32_RS10485	*icmH*	-1.993482214	5.15E-06
GBN32_RS10490	*tssH*	-1.703001067	5.70E-05
GBN32_RS10500	*tagO*	-1.665209632	9.49E-05
GBN32_RS10505	*tssA*	-1.872285451	1.24E-05
GBN32_RS10510	*tssM*	-1.975147033	3.76E-06
GBN32_RS12635	*lafU*	-1.548697386	0.000371641
GBN32_RS12640	*lafT*	-3.059486821	2.32E-07
GBN32_RS12645	*fliK$_L$*	-1.592091898	0.000183264
GBN32_RS12650	*fliL$_L$*	-2.217120059	1.01E-06

GBN32_RS12655	*lafX*	-1.65570496	1.61E-27
GBN32_RS12660	*fliS*$_L$	-1.724957778	0.000197853
GBN32_RS12665	*fliD*$_L$	-3.372997601	9.24E-13
GBN32_RS12670	*fliC*$_L$	-11.65570496	1.06E-32
GBN32_RS12685	*flgL*$_L$	-1.799941713	3.58E-05
GBN32_RS12685	*flgK*$_L$	-2.488462053	6.91E-08
GBN32_RS12745	*flgM*$_L$	-1.766325649	3.35E-05
GBN32_RS12750	*flgN*$_L$	-1.795228877	2.59E-05
GBN32_RS12825	*glmU*	-1.661065694	8.38E-05
GBN32_RS03520	*GBN32_RS03520*	2.044121974	0.000471263
GBN32_RS03685	*GBN32_RS03685*	4.665913802	4.10E-22
GBN32_RS03690	*GBN32_RS03690*	2.659478376	3.56E-09
GBN32_RS03695	*iolD*	4.991373208	2.84E-24
GBN32_RS03705	*iolG*	2.277114827	1.18E-07
GBN32_RS03710	*GBN32_RS03710*	6.407278876	6.74E-33
GBN32_RS03715	*GBN32_RS03715*	4.276571451	3.39E-19
GBN32_RS03720	*GBN32_RS03720*	3.431410113	1.70E-13
GBN32_RS03730	*iolC*	5.967167331	2.81E-31
GBN32_RS03735	*iolE*	4.629058728	2.82E-22
GBN32_RS03740	*iolB*	5.567167528	4.26E-28
GBN32_RS05930	*GBN32_RS05930*	1.592919635	0.000284802
GBN32_RS15940	*iscR*	1.594626611	0.000178341

Gene id：Gene number

log2FoldChange: The ratio of the gene expression levels of the treatment to control groups then taken as the logarithm of base 2

Pvalue: The p-value of the significance test

Fig. S1

A **B**

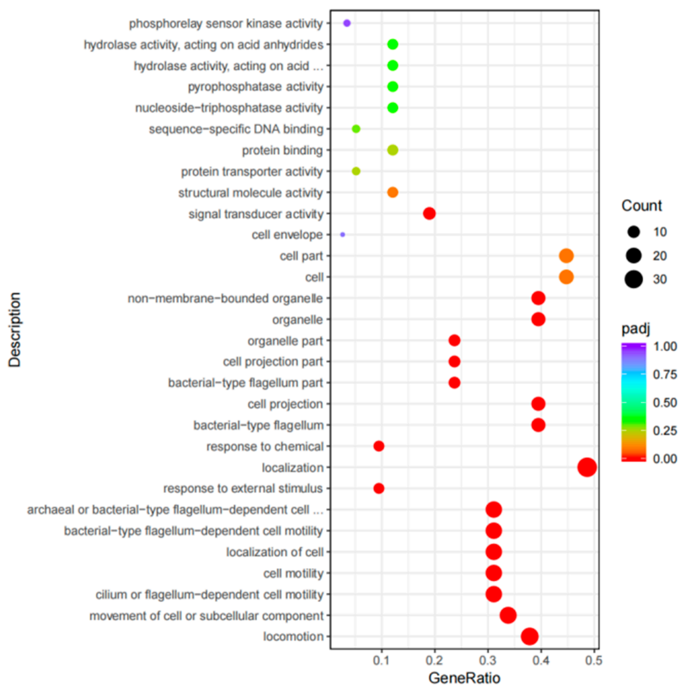
phosphorelay sensor kinase activity
hydrolase activity, acting on acid anhydrides
hydrolase activity, acting on acid ...
pyrophosphatase activity
nucleoside−triphosphatase activity
sequence−specific DNA binding
protein binding
protein transporter activity
structural molecule activity
signal transducer activity
cell envelope
cell part
cell
non−membrane−bounded organelle
organelle
organelle part
cell projection part
bacterial−type flagellum part
cell projection
bacterial−type flagellum
response to chemical
localization
response to external stimulus
archaeal or bacterial−type flagellum−dependent cell ...
bacterial−type flagellum−dependent cell motility
localization of cell
cell motility
cilium or flagellum−dependent cell motility
movement of cell or subcellular component
locomotion
Description
0.1
0.2
0.3
0.4
0.5
GeneRatio
Count
10
20
30
padj
1.00
0.75
0.50
0.25
0.00

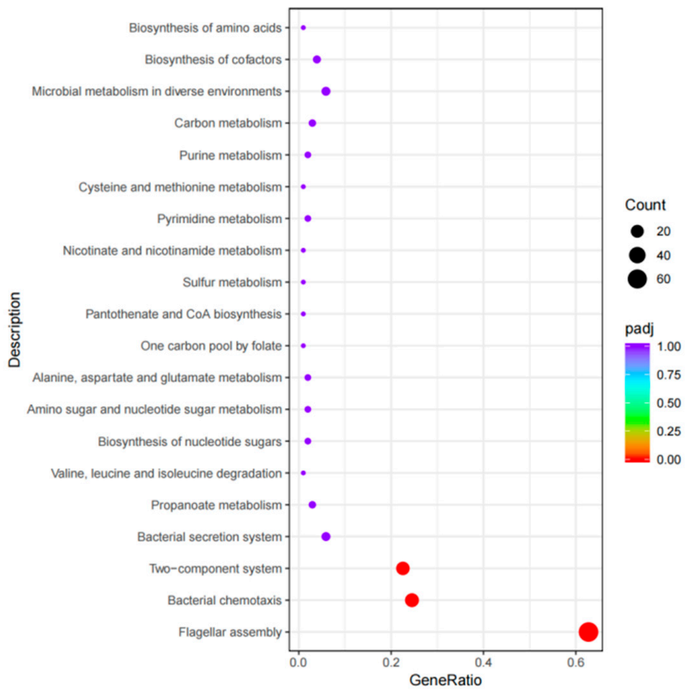
Biosynthesis of amino acids
Biosynthesis of cofactors
Microbial metabolism in diverse environments
Carbon metabolism
Purine metabolism
Cysteine and methionine metabolism
Pyrimidine metabolism
Nicotinate and nicotinamide metabolism
Sulfur metabolism
Pantothenate and CoA biosynthesis
One carbon pool by folate
Alanine, aspartate and glutamate metabolism
Amino sugar and nucleotide sugar metabolism
Biosynthesis of nucleotide sugars
Valine, leucine and isoleucine degradation
Propanoate metabolism
Bacterial secretion system
Two−component system
Bacterial chemotaxis
Flagellar assembly
Description
0.0
0.2
0.4
0.6
GeneRatio
Count
20
40
60
padj
1.00
0.75
0.50
0.25
0.00

C

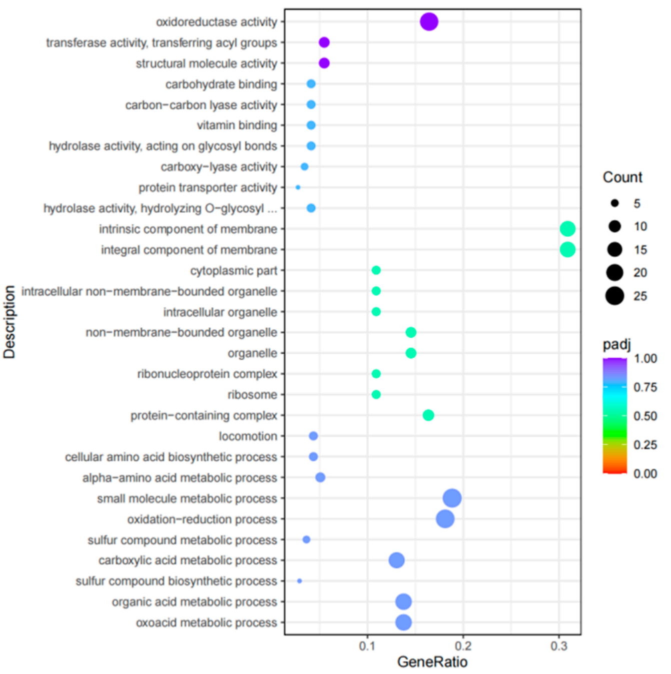
oxidoreductase activity
transferase activity, transferring acyl groups
structural molecule activity
carbohydrate binding
carbon−carbon lyase activity
vitamin binding
hydrolase activity, acting on glycosyl bonds
carboxy−lyase activity
protein transporter activity
hydrolase activity, hydrolyzing O−glycosyl ...
intrinsic component of membrane
integral component of membrane
cytoplasmic part
intracellular non−membrane−bounded organelle
intracellular organelle
non−membrane−bounded organelle
organelle
ribonucleoprotein complex
ribosome
protein−containing complex
locomotion
cellular amino acid biosynthetic process
alpha−amino acid metabolic process
small molecule metabolic process
oxidation−reduction process
sulfur compound metabolic process
carboxylic acid metabolic process
sulfur compound biosynthetic process
organic acid metabolic process
oxoacid metabolic process
Description
0.1
0.2
0.3
GeneRatio
Count
5
10
15
20
25
padj
1.00
0.75
0.50
0.25
0.00

D

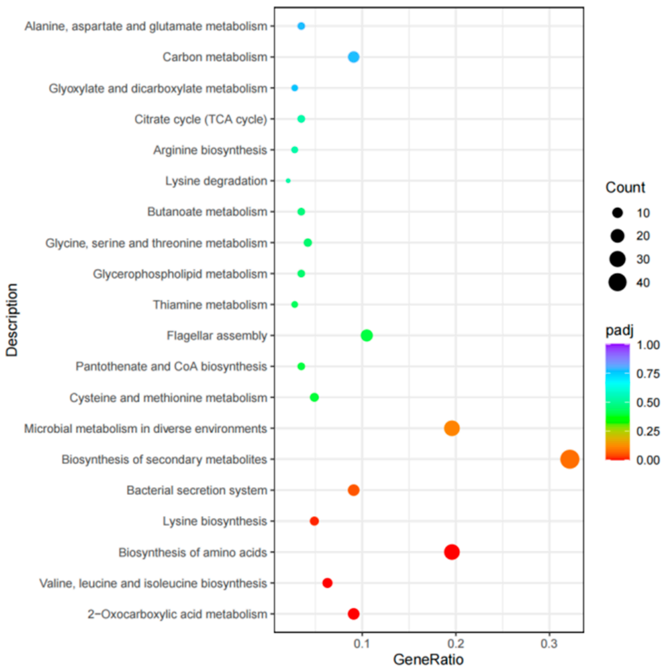
Alanine, aspartate and glutamate metabolism
Carbon metabolism
Glyoxylate and dicarboxylate metabolism
Citrate cycle (TCA cycle)
Arginine biosynthesis
Lysine degradation
Butanoate metabolism
Glycine, serine and threonine metabolism
Glycerophospholipid metabolism
Thiamine metabolism
Flagellar assembly
Pantothenate and CoA biosynthesis
Cysteine and methionine metabolism
Microbial metabolism in diverse environments
Biosynthesis of secondary metabolites
Bacterial secretion system
Lysine biosynthesis
Biosynthesis of amino acids
Valine, leucine and isoleucine biosynthesis
2−Oxocarboxylic acid metabolism
Description
0.1
0.2
0.3
GeneRatio
Count
10
20
30
40
padj
1.00
0.75
0.50
0.25
0.00

E

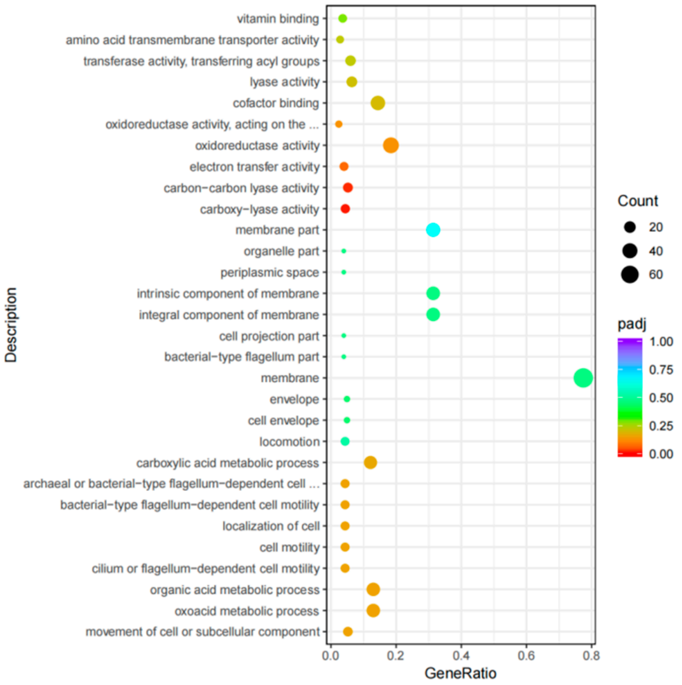
vitamin binding
amino acid transmembrane transporter activity
transferase activity, transferring acyl groups
lyase activity
cofactor binding
oxidoreductase activity, acting on the ...
oxidoreductase activity
electron transfer activity
carbon−carbon lyase activity
carboxy−lyase activity
membrane part
organelle part
periplasmic space
intrinsic component of membrane
integral component of membrane
cell projection part
bacterial−type flagellum part
membrane
envelope
cell envelope
locomotion
carboxylic acid metabolic process
archaeal or bacterial−type flagellum−dependent cell ...
bacterial−type flagellum−dependent cell motility
localization of cell
cell motility
cilium or flagellum−dependent cell motility
organic acid metabolic process
oxoacid metabolic process
movement of cell or subcellular component
Description
0.0
0.2
0.4
0.6
0.8
GeneRatio
Count
20
40
60
padj
1.00
0.75
0.50
0.25
0.00

F

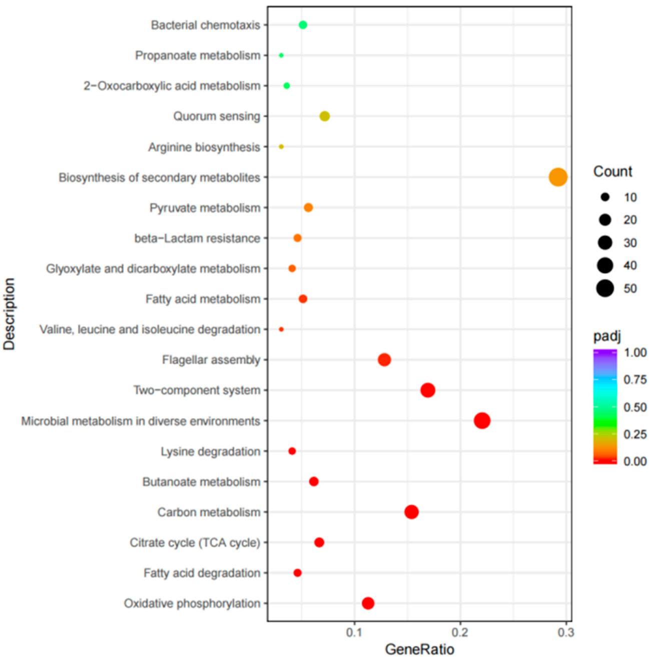
Bacterial chemotaxis
Propanoate metabolism
2−Oxocarboxylic acid metabolism
Quorum sensing
Arginine biosynthesis
Biosynthesis of secondary metabolites
Pyruvate metabolism
beta−Lactam resistance
Glyoxylate and dicarboxylate metabolism
Fatty acid metabolism
Valine, leucine and isoleucine degradation
Flagellar assembly
Two−component system
Microbial metabolism in diverse environments
Lysine degradation
Butanoate metabolism
Carbon metabolism
Citrate cycle (TCA cycle)
Fatty acid degradation
Oxidative phosphorylation
Description
0.1
0.2
0.3
GeneRatio
Count
10
20
30
40
50
padj
1.00
0.75
0.50
0.25
0.00

G **H**

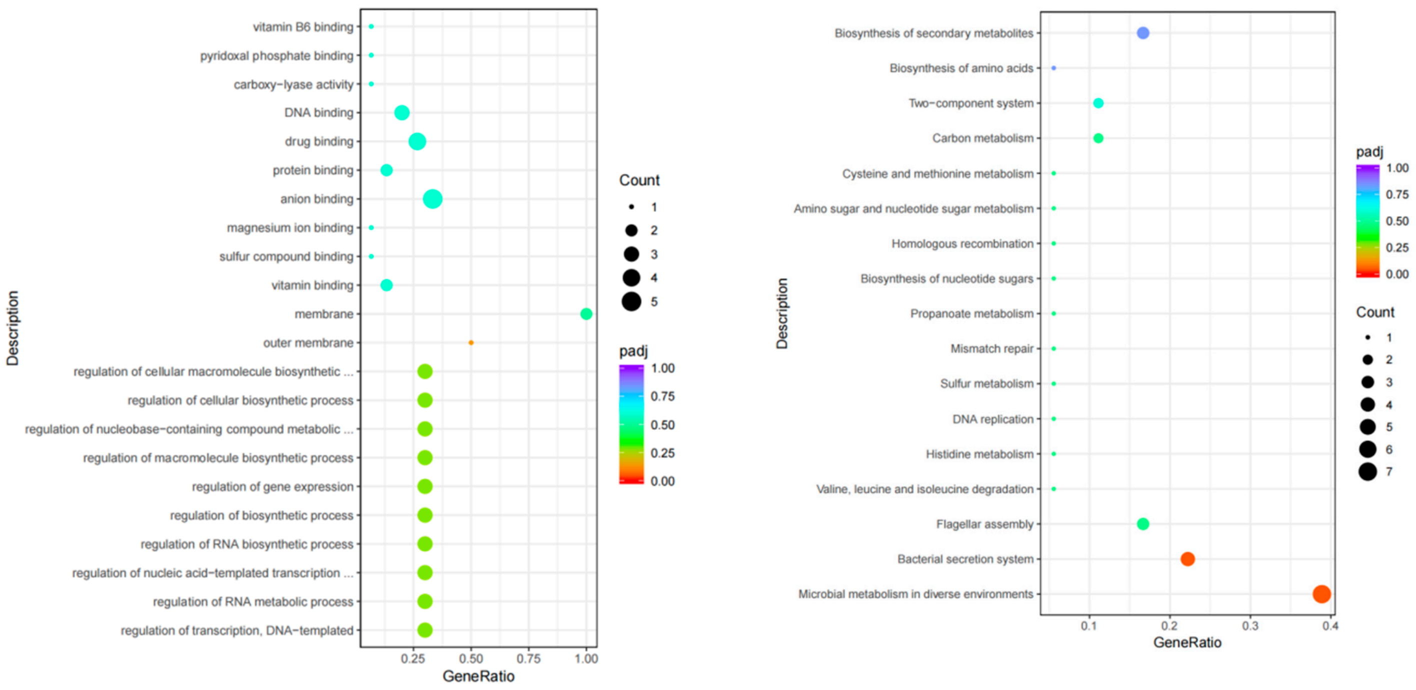

Fig. S1 GO (A) and KEGG (B) enrichment of DEGs in the Δ*flaK* transcriptome. GO (C) and KEGG (D) enrichment of DEGs in the Δ*flaM* transcriptome. GO (E) and KEGG (F) enrichment of DEGs in the Δ*fliA* transcriptome. GO (G) and KEGG (H) enrichment of DEGs in the Δ*fliA*$_L$ transcriptome, the GeneRatio refers to the ratio of the number of DEGs in the pathway and the number of all annotated genes in the pathway.

Figure S2

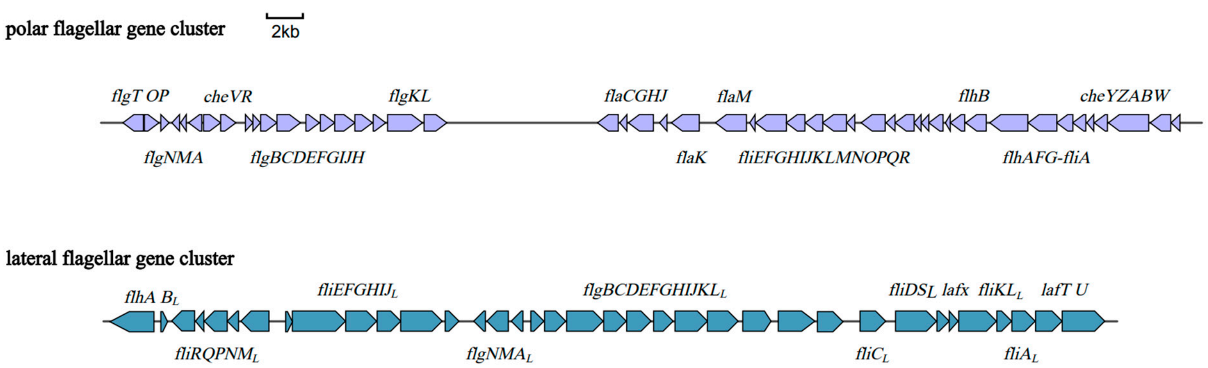

Figure S2 *P. shigelloides* flagella clusters, both polar and lateral.

Figure S3

A

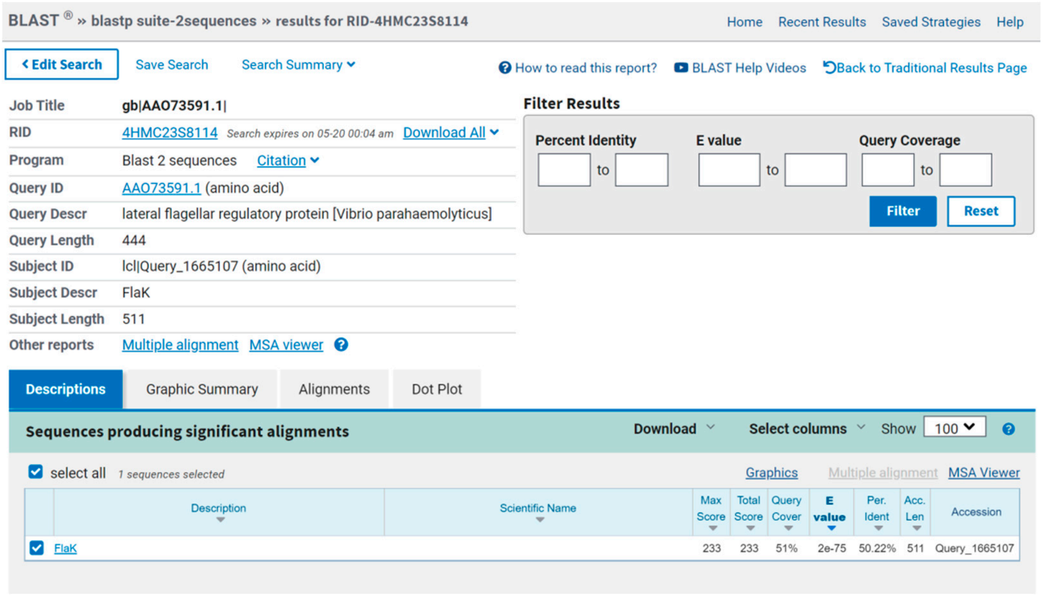

B

Score	Expect	Method	Identities	Positives	Gaps
233 bits(594)	2e-75	Compositional matrix adjust.	116/231(50%)	161/231(69%)	2/231(0%)

```
Query  125  NIVAESWRSKQVLQLAHRAACTNASVLITGESGTGKEVLARYVHEHSPRINGPFVAVNCA  184
            ++V  S   + V ++   + A T+A+VLI GESGTGKEV+AR +H +S R  GPFV +NC
Sbjct  138  SLVGRSHAVQNVRRMIEQVAPTDANVLILGESGTGKEVVARNIHYYSARREGPFVPLNCG  197

Query  185  AIPESMLEAVLFGHVKGAFTGATNSQSGKFEEANGGTILLDEIGEMSPAVQAKLLRVLQE  244
            AIP  +LE+ LFGH KGAFTGA +++ G+FE A GGT+ LDEIG+M  ++Q KLLRVLQE
Sbjct  198  AIPAELLESELFGHEKGAFTGAISARKGRFELAEGGTLFLDEIGDMPMSMQVKLLRVLQE  257

Query  245  REVERVGSHKAIKLDIRVIAATNKDLREEVQKGTFREDLYYRLDVLPLHWPPLRERKEDI  304
            R  ERVGS+K I+ D+RVIAAT++L E +  G FR+DLYYRL+V P+  P LRER EDI
Sbjct  258  RCFERVGSNKTIRADVRVIAATHRNLEEMIAGGEFRQDLYYRLNVFPIDVPALRERAEDI  317

Query  305  --LPISQFFIEKYQDSSRCHLSQDAISALSQYHWPGNIRELENVIQRALVM  353
              L          + +    H +   A+ +L +Y W GN+REL N+++R +++
Sbjct  318  PLLLQELLSRLTAEGARSVHFTPRALHSLCEYSWSGNVRELANLVERLVIL  368
```

Figure S3 Amino acid homology similarity (A) and alignment results (B) between LafK of *V. parahaemolyticus* and FlaK of *P. shigelloides*. Query represents the amino acid sequence of *V. parahaemolyticus*'s LafK (GenBank: AAO73591.1), while Sbjct represents *P. shigelloides*' FlaK (GenBank: EON89963.1).

Figure S4

A

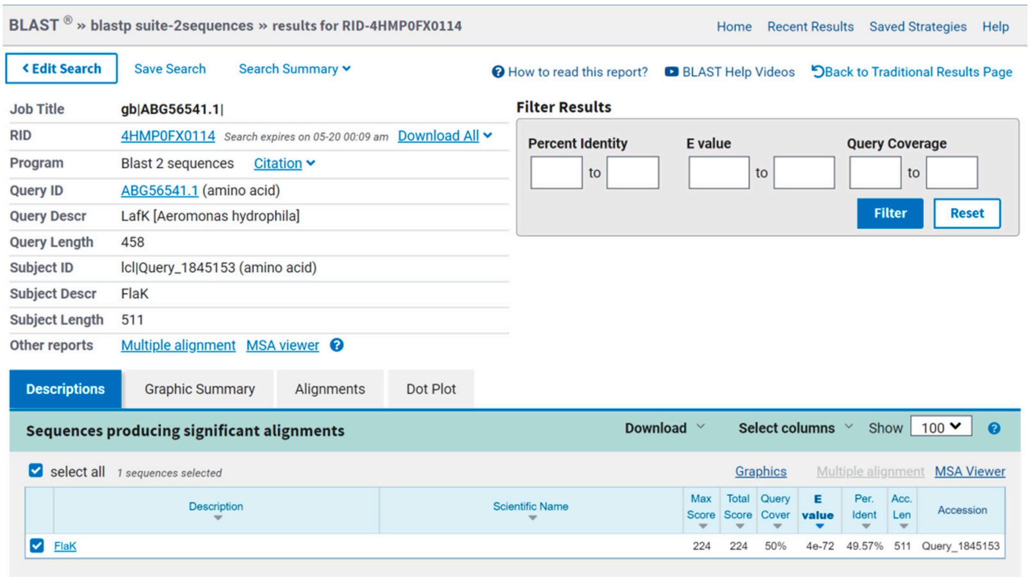

B

Score	Expect	Method	Identities	Positives	Gaps
228 bits(580)	3e-73	Compositional matrix adjust.	115/232(50%)	158/232(68%)	4/232(1%)

```
Query  139  NLIATSAMTLQVLQLARRAAKTSATILIRGESGTGKERLARYIHDMSNRASKPFIAINCA  198
            +L+  S      V ++   + A T A +LI GESGTGKE +AR IH  S R    PF+ +NC
Sbjct  138  SLVGRSHAVQNVRRMIEQVAPTDANVLILGESGTGKEVVARNIHYYSARREGPFVPLNCG  197

Query  199  AIPESMLESILFGHNKGAFTGATGTQPGKFELAQGGTLLLDEISELPLSLQAKLLRVLQE  258
            AIP  +LES LFGH KGAFTGA     + G+FELA+GGTL LDEI  ++P+S+Q KLLRVLQE
Sbjct  198  AIPAELLESELFGHEKGAFTGAISARKGRFELAEGGTLFLDEIGDMPMSMQVKLLRVLQE  257

Query  259  KEVERLGSHQTIALDVRIIAASNKDIRTLVAVGQFREDLFYRLDVLPMSWPALRERPDDI  318
            +  ER+GS++TI  DVR+IAA+++++  ++A G+FR+DL+YRL+V P+  PALRER +DI
Sbjct  258  RCFERVGSNKTIRADVRVIAATHRNLEEMIAGGEFRQDLYYRLNVFPIDVPALRERAEDI  317

Query  319  LPLAEYFIERHAPQQGYR---LSESACQQLLAYGWPGNVRELDNVIQRSLIM  367
             PL     +       +G R     +  A    L   Y W GNVREL N+++R +I+
Sbjct  318  -PLLLQELLSRLTAEGARSVHFTPRALHSLCEYSWSGNVRELANLVERLVIL  368
```

Figure S4 Amino acid homology similarity (A) and alignment results (B) between LafK of *A. hydrophila* and FlaK of *P. shigelloides*. Query represents the amino acid sequence of *A. hydrophila*'s LafK (NCBI Reference Sequence: WP_346814955.1), while Sbjct represents *P. shigelloides*' FlaK (GenBank: EON89963.1).

Figure S5

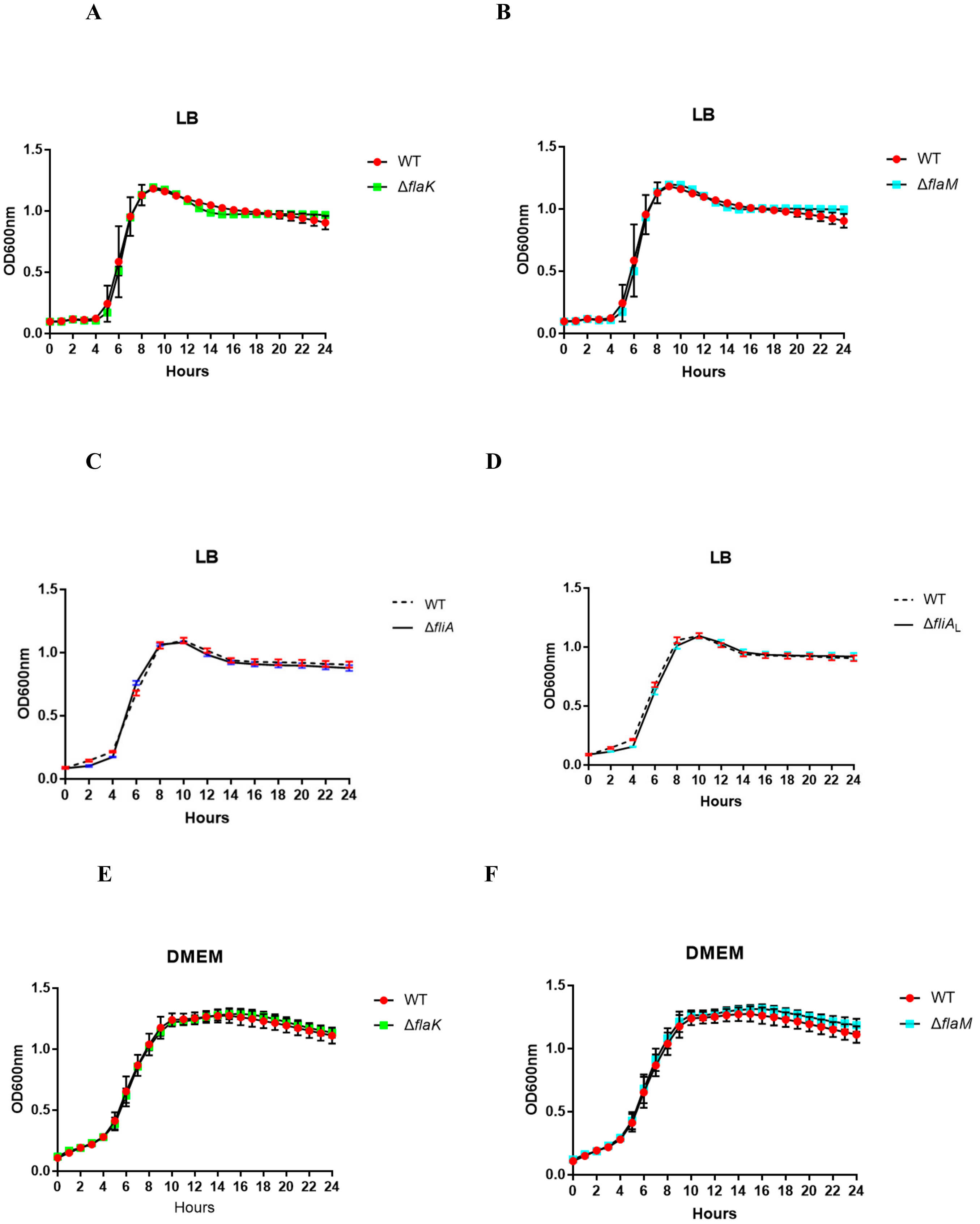
A
B
LB
OD600nm
Hours
WT
ΔflaK
ΔflaM
C
D
LB
WT
ΔfliA
ΔfliA_L
E
F
DMEM
WT
ΔflaK
ΔflaM

G **H**

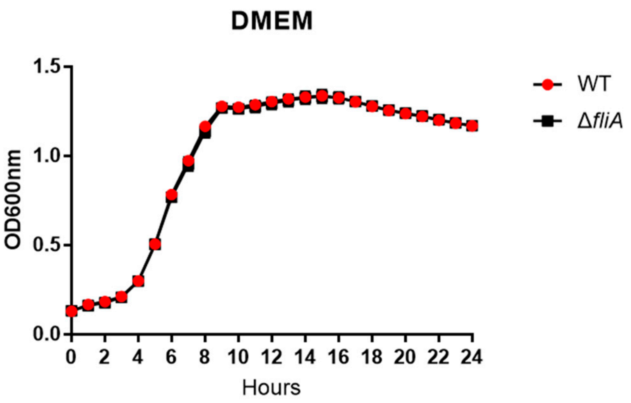

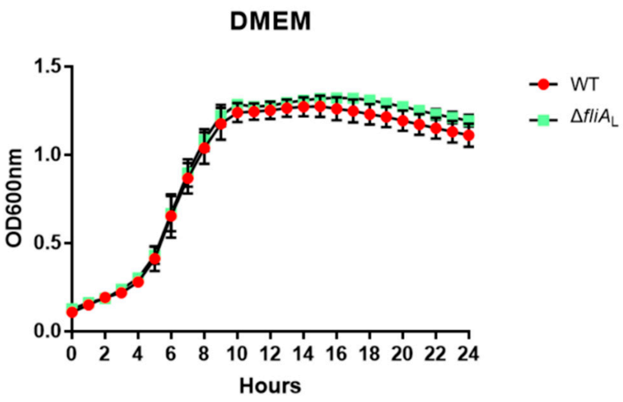

Figure S5 The dynamic growth experiment for the WT and Δ*flaK* strains was carried out in LB medium (A) and DMEM (E). The dynamic growth experiment for the WT and Δ*flaM* strains was carried out in LB medium (B) and DMEM (F). The dynamic growth experiment for the WT and Δ*fliA* strains was carried out in LB medium (C) and DMEM (G). The dynamic growth experiment for the WT and Δ*fliA$_L$* strains was carried out in LB medium (D) and DMEM (H).
